# Crystal structures of [Mn(bdc)(Hspar)_2_(H_2_O)_0.25_]·2H_2_O containing MnO_6+1_ capped trigonal prisms and [Cu(Hspar)_2_](bdc)·2H_2_O containing CuO_4_ squares (Hspar = sparfloxacin and bdc = benzene-1,4-di­carboxyl­ate)

**DOI:** 10.1107/S205698901502424X

**Published:** 2016-01-01

**Authors:** Zhe An, Jing Gao, William T. A. Harrison

**Affiliations:** a School of Chemistry and Life Science, Guangdong University of Petrochemical Technology, Maoming 525000, People’s Republic of China; b Department of Pharmacy, Mudanjiang Medical University, Heilongjiang 157011, People’s Republic of China; c Department of Chemistry, University of Aberdeen, Meston Walk, Aberdeen AB24 3UE, Scotland

**Keywords:** crystal structure, sparfloxacin, trigonal prismatic geometry, manganese, copper, mol­ecular salt

## Abstract

The syntheses and crystal structures of 0.25-aqua­(benzene-1,4-di­carboxyl­ato-κ^2^ *O*,*O*′)bis­(sparfloxacin-κ^2^ *O*,*O*′)manganese(II) dihydrate, [Mn(C_8_H_4_O_4_)(C_19_H_22_F_2_N_4_O_3_)_2_(H_2_O)_0.25_]·2H_2_O or [Mn(bdc)(Hspar)_2_(H_2_O)_0.25_]·2H_2_O, (I), and bis­(sparfloxacin-κ^2^ *O*,*O*′)copper(II) benzene-1,4-di­carboxyl­ate dihydrate, [Cu(C_19_H_22_F_2_N_4_O_3_)_2_](C_8_H_4_O_4_)·2H_2_O or [Cu(Hspar)_2_](bdc)·2H_2_O, (II), are reported (Hspar = sparfloxacin and bdc = benzene-1,4-di­carboxyl­ate). The Mn^2+^ ion in (I) is coordinated by two *O*,*O*′-bidentate Hspar neutral mol­ecules (which exist as zwitterions) and an *O*,*O*′-bidentate bdc dianion to generate a distorted MnO_6_ trigonal prism. A very long bond [2.580 (12) Å] from the Mn^2+^ ion to a 0.25-occupied water mol­ecule projects through a square face of the prism. In (II), the Cu^2+^ ion lies on a crystallographic inversion centre and a CuO_4_ square-planar geometry arises from its coordination by two *O*,*O*′-bidentate Hspar mol­ecules. The bdc dianion acts as a counter-ion to the cationic complex and does not bond to the metal ion. The Hspar ligands in both (I) and (II) feature intra­molecular N—H⋯O hydrogen bonds, which close *S*(6) rings. In the crystals of both (I) and (II), the components are linked by N—H⋯O, O—H⋯O and C—H⋯O hydrogen bonds, generating three-dimensional networks.

## Abstract 2

The Mn^2+^ ion in [Mn(bdc)(Hspar)_2_(H_2_O)_0.25_]·2H_2_O (Hspar = sparfloxacin and bdc = benzene-1,4-di­carboxyl­ate) is coordinated by two *O*,*O*′-bidentate Hspar neutral mol­ecules (which exist as zwitterions) and an *O*,*O*′-bidentate bdc dianion to generate a distorted MnO_6_ trigonal prism. In [Cu(Hspar)_2_](bdc)·2H_2_O,the Cu^2+^ ion lies on a crystallographic inversion centre and a CuO_4_ square-planar geometry arises from its coordination by two *O*,*O*′-bidentate Hspar mol­ecules. The bdc dianion acts as a counter-ion to the cationic complex and does not bond to the metal ion.

## Chemical context   

Sparfloxacin, C_19_H_22_F_2_N_4_O_3_ (Hspar; systematic name: 5-amino-1-cyclo­propyl-7-[(3R*,5S*)(3,5-di­methyl­piperazin-1-yl]-6,8-di­fluoro-4-oxo-quinoline-3-carb­oxy­lic acid) (Miyamoto *et al.*, 1990 ▸; Qadri *et al.*, 1992 ▸) is a member of the quinolone (Andersson & MacGowan, 2003 ▸) family of anti­biotics; other well-known examples of this group of compounds include ciprofloxacin (C_17_H_18_FN_3_O_3_) and enroflaxacin (C_19_H_22_FN_3_O_3_). As well as their biological significance, this class of compounds is of inter­est in coordination chemistry due to their potential to act as multi-dentate and bridging ligands in the construction of mononuclear and dinuclear complexes (An *et al.*, 2008 ▸, 2010 ▸) and coordination polymers (Xiao *et al.*, 2005 ▸; Yu *et al.*, 2009 ▸).

As well as hydrated Hspar, which occurs in the crystal in its zwitterionic form, *i.e.* proton transfer from the –CO_2_H carb­oxy­lic acid group to the remote secondary amine moiety of the piperazine ring (Sivalakshmidevi *et al.*, 2000 ▸), the crystal structures of its anionic (spar^−^) complexes with nickel (Skyrianou *et al.*, 2009 ▸), copper (Efthimiadou *et al.*, 2006 ▸) and zinc (Tarushi *et al.*, 2011 ▸) have been reported. Hydrated mol­ecular salts of the H_2_spar^+^ cation (*i.e.* containing both –CO_2_H and NH_2_^+^ groups) with BF_4_^−^ (Shingnapurkar *et al.*, 2007 ▸) and SO_4_^2−^ counter-ions (Li *et al.*, 2011 ▸) are known. As part of our own studies in this area, we have recently described the structure of [Cd(spar)_2_]·H_2_O (An *et al.*, 2012 ▸), a one-dimensional coordination polymer in which chains of CdO_6_ octa­hedra bridged by the spar^−^ species are found.
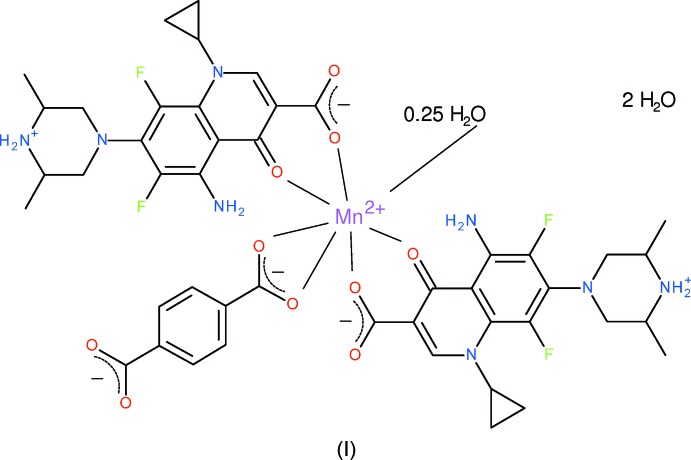
 
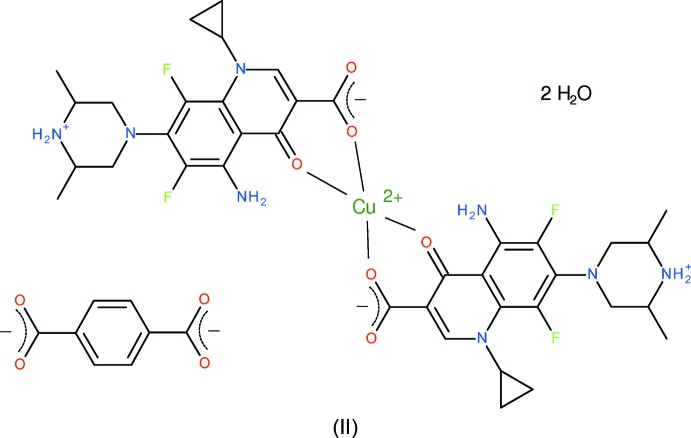


As a continuation of these studies, we now describe the syntheses and crystal structures of the title mixed-ligand complexes [Mn(bdc)(Hspar)_2_(H_2_O)_0.25_]·2H_2_O (I) and [Cu(Hspar)_2_](bdc)·2H_2_O (II) (bdc = benzene-1,4-di­carboxyl­ate, C_8_H_4_O_4_^2−^).

## Structural commentary   

### Compound (I)   

**Figure 1:**
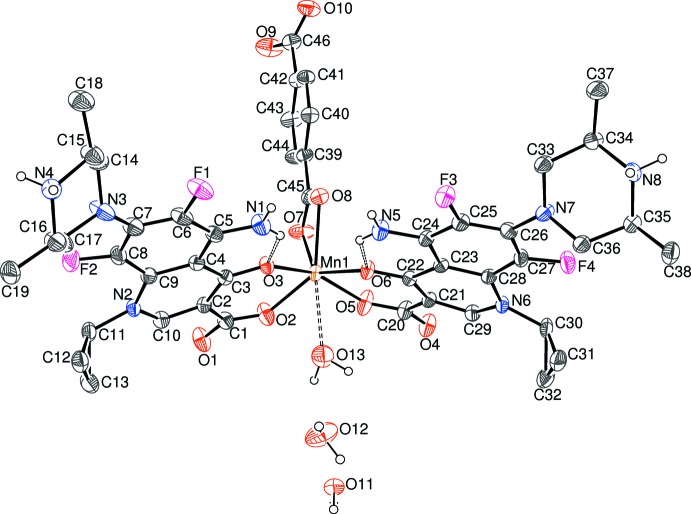
The mol­ecular structure of (I), showing 50% displacement ellipsoids. H atoms bound to C atoms have been omitted for clarity and hydrogen bonds and the long Mn1⋯O13 contact are shown as double-dashed lines.

Compound (I) is a hydrated neutral mononuclear complex: the asymmetric unit contains an Mn^2+^ cation, two neutral, zwitterionic Hspar mol­ecules, a bdc dianion and three water mol­ecules, one of which, O13, was modelled with a site occupancy factor of 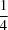 (Fig. 1 ▸).

**Figure 2:**
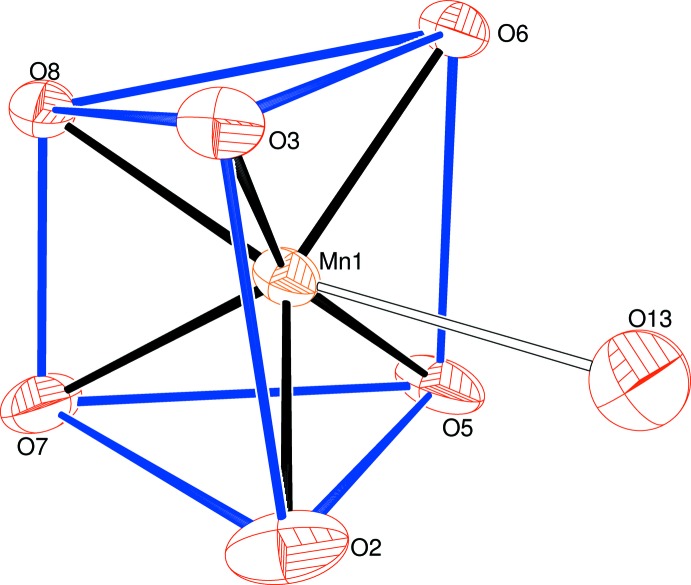
Detail of (I) showing the capped trigonal prismatic coordination of the metal ion.

The manganese ion in (I) is coordinated by two bidentate Hspar mol­ecules, with the quinoline O atom and its *syn*-carboxyl­ate O atom (O3 and O2, respectively, in the C1-containing mol­ecule and O6 and O5, respectively, in the C20-mol­ecule) serving as the donor atoms, which generates a six-membered chelate ring in each case, with O—Mn—O bite angles of 81.86 (8) and 82.05 (8)°, respectively. The metal coordination sphere also features an *O*,*O*-bidentate bdc dianion and a very long [2.580 (12) Å] Mn—O bond to the partly occupied O13 water mol­ecule. Together, these lead to a distorted MnO_6+1_ trigonal–prismatic polyhedron (Table 1 ▸) with the Mn—O*w* bond capping through the square face defined by the two Hspar ligands (Fig. 2 ▸). The mean Mn—O separation of 2.137 Å for the Hspar bonds is significantly shorter than the mean of the Mn—O (bdc) bonds of 2.297 Å and the bond-valence sum (BVS) (Brown & Altermatt, 1985 ▸) for the metal ion for the six shorter bonds is 1.89 (expected value = 2.00). If the seventh bond to O13 is added, the manganese BVS increases to 1.99.

The conformation of the –O2–C1–C2–C3–O3–Mn1– chelate ring approximates to a shallow envelope with the metal atom as the flap, displaced by −0.222 (4) Å from the mean plane of the ligand atoms (r.m.s. deviation = 0.022 Å). The –O5–C20–C21–C22–O6–Mn1– ring can be described in the same way, with Mn1 displaced by 0.128 (4) Å from the other atoms (r.m.s. deviation = 0.019 Å). The dihedral angle between the near-planar segments of the chelate rings is 29.74 (13)°. Both Hspar mol­ecules are orientated in the same sense with respect to the metal ion, with the NH_2_ groups mutually *syn*.

The capped trigonal–prismatic geometry of the MnO_6+1_ grouping is unusual and calls for some further comment: the dihedral angle between the top (O3/O6/O8) and bottom (O2/O5/O7) triangular faces of the prism is 14.40 (11)°, which is largely due to the O7⋯O8 edge of the prism (the two O atoms of the bdc dianion) being much shorter [2.174 (3) Å] than the O2⋯O3 [2.799 (3) Å] and O5⋯O6 [2.802 (3) Å] edges, which correspond to the C1- and C-20 Hspar mol­ecules, respectively. The metal atom is displaced from the top and bottom faces of the prism by −1.2513 (14) and 1.3670 (12) Å, respectively. The degree of twist of the prism may be estimated from the pseudo torsion angles involving the centroids of the triangular faces (denoted *X*1 for the O3/O6/O8 face and *X*2 for the O2/O5/O7 face) and the pairs of atoms forming the edges of the prism: values of *X*1⋯O7⋯O8⋯*X*2 (–14.6), *X*1⋯O5⋯O6⋯*X*2 (–11.2) and *X*1⋯O2⋯O3⋯*X*2 (–8.5°) arise. These angles would be zero for a perfect triangular prism.

The most important geometrical features of the first Hspar mol­ecule (containing C1) are as follows: the C1—O1 and C1—O2 bond lengths of 1.251 (4) and 1.256 (4) Å, respectively, are typical for a delocalized carboxyl­ate group and the dihedral angle between C1/O1/O2 and the adjacent N2-containing ring (r.m.s. deviation = 0.045 Å) is 8.6 (8)°. The dihedral angle between the cyclo­propane ring and the N2 ring is 67.5 (3)°. The N2 bond-angle sum of 359.8° is consistent with a bonding model of *sp* ^2^ hybridization for this atom. The dihedral angle between the N2 ring and the C5 ring (r.m.s. deviation = 0.028 Å), which are fused at the C4—C9 bond, is 7.9 (2)°, indicating a substantial puckering to the quinolone system. The piperazinium ring adopts a typical chair conformation with the exocyclic N—C_q_ (q = quinolone) bond in an equatorial orientation. The dihedral angle between the four C atoms that form the ‘seat’ of the chair and the C5 ring is 60.3 (2)°. There was some suggestion that atoms C14 and C17 of this ring are positionally disordered, but refinements that attempted to model this effect were inconclusive.

The second Hspar mol­ecule (containing C20) has a broadly similar geometry: the C20—O4 and C20—O5 bond lengths are 1.254 (4) and 1.257 (4) Å, respectively, and the dihedral angle between C20/O4/O5 and the N6 ring (r.m.s. deviation = 0.050 Å) is 8.8 (7)°. The dihedral angle between the N6 (bond-angle sum = 359.7°) ring and the pendent three-membered ring is 69.8 (2)°. The N6 and C24 rings (r.m.s. deviation for the latter = 0.020 Å), fused at the C23—C28 bond, are tilted by 8.1 (2)°. The piperazine ring adopts a chair conformation and the dihedral angle between the chair seat and the C24 ring is 58.71 (9)°. Each Hspar mol­ecule features an intra­molecular N—H⋯O hydrogen bond (Table 2 ▸), which closes an *S*(6) ring. The C45/O7/O8 and C46/O9/O10 carboxyl­ate groups of the bdc dianion are rotated by 3.90 (7) and 25.28 (14)°, respectively with respect to the central ring plane. The O7—Mn1—O8 bite angle is 56.58 (8)°.

### Compound (II)   

**Figure 3:**
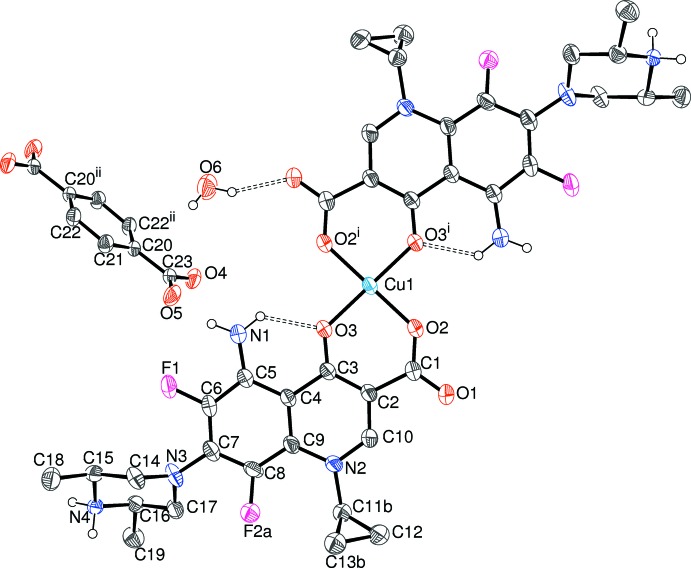
The mol­ecular structure of (II) showing 50% probability displacement ellipsoids. Only one orientation of the disordered cyclo­propyl ring is shown. Hydrogen bonds are shown as double-dashed lines. [Symmetry codes: (i) 1 − *x*, −*y*, 1 − *z*; (ii) −*x*, −*y*, −*z*.]

Compound (II) can be regarded as a hydrated mol­ecular salt: the asymmetric unit contains a Cu^2+^ cation lying on a crystallographic inversion centre, a neutral, zwitterionic, Hspar mol­ecule, half a bdc dianion and a water mol­ecule of crystallization (Fig. 3 ▸).

The copper ion in (II) is coordinated by two *O*,*O*-bidentate Hspar mol­ecules in the usual bonding mode of quinoline O atom + *syn*-carboxyl­ate O atom (O3 and O2, respectively) with a bite angle of 93.24 (8)°, which generates a six-membered chelate ring. The result is a CuO_4_ square-planar coordination polyhedron (Table 3 ▸) with a mean Cu—O separation of 1.898 Å. There are no atoms in possible axial sites within 3.5 Å of the metal ion. The –O2–C1–C2–C3–O3–Cu1– chelate ring is a shallow envelope, with the metal atom displaced by 0.124 (3) Å from the mean plane of the almost planar ligand atoms (r.m.s. deviation = 0.023 Å).

In the Hspar mol­ecule, the C1—O1 and C1—O2 bond lengths are distinctly different at 1.226 (4) Å and 1.283 (4) Å, respectively, unlike the situation in (I), where they are almost the same length. The dihedral angle between the C1/O1/O2 grouping in (II) and its attached ring is 6.2 (5)° and the dihedral angle between the fused rings of the quinolone system is 3.2 (2)°. The cyclo­propane ring in (II) is disordered over two orientations in a 0.670 (8): 0.330 (8) ratio. The piperazine ring adopts a chair conformation as usual, and N4 (the secondary amine group) is protonated. The dihedral angle between the four carbon atoms forming the ‘seat’ of the chair and the F-bearing aromatic ring is 63.77 (10)°.

In the bdc dianion, the C23/O4/O5 carboxyl­ate group is rotated by 2.7 (6)° with respect to the aromatic ring plane. The C23—O4 and C23—O5 bond lengths of 1.244 (4) and 1.253 (4) Å, respectively, are consistent with the approximately equal delocalization of the negative charge over both C—O bonds.

## Supra­molecular features   

In the crystal of (I), a number of N—H⋯O, O—H⋯O and weak C—H⋯O hydrogen bonds (Table 2 ▸) link the components into a three-dimensional network. A short C—H⋯π inter­action is also observed.

In (II), the packing is consolidated by N—H⋯O, O—H⋯O and weak C—H⋯O hydrogen bonds (Table 4 ▸), resulting in a three-dimensional network.

## Database survey   

So far as a search of the Cambridge Structural Database (Groom & Allen, 2014 ▸) reveals, (I) is the first crystal structure of a complex containing Mn^2+^ ions and Hspar mol­ecules. The *O*,*O*-chelating mode of the Hspar mol­ecules is normal for other divalent transition metals (Skyrianou *et al.*, 2009 ▸; Efthimiadou *et al.*, 2006 ▸; Tarushi *et al.*, 2011 ▸), as is that of the *O*,*O*-bidentate bdc dianion for Mn^2+^ (*e.g.* Ma *et al.*, 2003 ▸), but the resulting trigonal–prismatic coordination geometry for the manganese ion in (I) is very unusual, although not unknown. An analogous structure is seen for [Mn(acac)_2_(bipy)] (acac = acetyl­acetonate, bipy = 2,2′-bi­pyridine; van Gorkum *et al.*, 2005 ▸), where an almost regular MnN_2_O_4_ trigonal prism occurs (*i.e.* there is no capping): as these authors note, the high-spin *d* ^5^ electronic configuration of Mn^2+^ is the ‘least unexpected’ to show a trigonal–prismatic geometry because it has no crystal-field stabilization energy, which normally favours octa­hedral over trigonal–prismatic geometry (Karpishin *et al.*, 1993 ▸). Based on DFT calculations, it was concluded that the trigonal–prismatic and octa­hedral geometries for [Mn(acac)_2_(bipy)] have almost the same energy and the trigonal–prismatic geometry is adopted in the crystal because of favourable packing inter­actions (van Gorkum *et al.*, 2005 ▸). The ligands in (I) are far bulkier and more flexible than acac or bipy and it is difficult to speculate on whether packing effects are equally important in establishing the capped trigonal–prismatic metal-ion coordination geometry in (I).

Compound (II) complements several previously studied Cu–sparfloxacin complexes including [Cu(spar)_2_]·2.8H_2_O (Efthimiadou *et al.*, 2006 ▸), in which centrosymmetric neutral Cu(spar)_2_ mol­ecules occur, compared to the centrosymmetric [Cu(Hspar)_2_]^2+^ cations seen here. In [Cu(H_2_spar)(H_2_O)(phen)]BF_4_·3H_2_O (phen = 1,10-phenanthroline; Shingnapurkar *et al.*, 2007 ▸), the metal ion is chelated by the *O*,*O*-bidentate H_2_spar^+^ cation (deprotonated at the carboxyl group and protonated at both the primary and secondary amine N atoms) and the *N*,*N*-bidentate phen ligand in a square-planar arrangement; the water mol­ecule completes the square-based pyramidal coordination polyhedron in the apical site. Finally, in the novel bimetallic complex [Cu_2_(spar)_4_]·4H_2_O (Shingnapurkar *et al.*, 2007 ▸), the Cu^2+^ ions are chelated by two spar^−^ anions in the basal plane, with a long apical Cu—N bond [2.463 (4) Å] arising from the –NH_2_ group of an adjacent spar^−^ anion generating a centrosymmetric, bimetallic assembly. It is thus notable that sparfloxacin can bind to Cu^2+^ ions in its anionic, neutral and cationic forms and we are continuing our explorations of these systems.

## Synthesis and crystallization   

To prepare (I), a mixture of Mn(CH_3_CO_2_)_2_·4H_2_O (0.25 mmol), sparfloxacin (0.5 mmol), 1,4-benzene­dicarb­oxy­lic acid (0.25 mmol), sodium hydroxide (1 mmol) and water (15 ml) was stirred for 30 minutes in air. The mixture was placed in a sealed 25 ml Teflon-lined hydro­thermal reactor and heated to 423 K for 72 h under autogenous pressure. Upon cooling, colourless prisms of (I) were recovered from the reaction by vacuum filtration and rinsing with water. Analysis calculated (found) (%) for C_46_H_52.5_MnF_4_N_8_O_12.25_: C 52.90 (52.63), H 5.07 (4.91), N 10.73 (10.58). IR (KBr, cm^−1^): *br*3420, *br*3300, *s*1633 (C=O pyridone), *s*1562 (CO_2_ *asym*), *s*1443, *s*1375 (CO_2_ *symm*), *s*1292, *w*1184, *m*819, *m*756, *m*686, *m*517 [IR assignments following Llinàs *et al.* (2008 ▸)].

Compound (II) was prepared by the same method with [Cu(CH_3_CO_2_)_2_]·H_2_O (0.25 mmol) used in place of the manganese acetate tetra­hydrate and the vessel heated to 413 K for 72 h. Upon cooling, green blocks of (II) were obtained from the reaction mixture. Analysis calculated (found) (%) for C_46_H_50_CuF_4_N_8_O_12_: C 52.80 (52.70), H 4.82 (4.72), N 10.71 (10.64). IR (KBr, cm^−1^): *br*3427, *br*3304, *s*1633 (C=O pyridone), s1556 (CO_2_ *asym*), *s*1435, *s*1358 (CO_2_ *symm*), *s*1294, *w*1182, *w*1012, *w*928, *w*814, *m*748, *m*527.

Both (I) and (II) appear to be indefinitely stable when stored in dry air.

## Refinement   

Crystal data, data collection and structure refinement details for (I) and (II) are summarized in Table 5 ▸. In (I), the O13 water mol­ecule is close to an inversion-generated clone and cannot be more than 50% occupied. Its site occupancy was refined and converged to close to 0.25: in the final cycles of refinement, it was fixed at 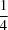. In (II), the pendant cyclo­propane group is disordered over two orientations in a 0.670 (8): 0.330 (8) ratio and one of the fluorine atoms is disordered over two sites in a 0.544 (11):0.456 (11) ratio. For both structures, the C-bound H atoms were geometrically placed and refined as riding atoms with the constraint *U*_iso_(H) = 1.2–1.5*U*_eq_(C) applied. The N- and O-bound H atoms were located in difference maps and refined as riding atoms in their as-found relative positions.

## Supplementary Material

Crystal structure: contains datablock(s) I, II, global. DOI: 10.1107/S205698901502424X/sj5492sup1.cif

Structure factors: contains datablock(s) I. DOI: 10.1107/S205698901502424X/sj5492Isup2.hkl

Structure factors: contains datablock(s) II. DOI: 10.1107/S205698901502424X/sj5492IIsup3.hkl

CCDC references: 932077, 932078

Additional supporting information:  crystallographic information; 3D view; checkCIF report

## Figures and Tables

**Table 1:** Selected geometric parameters (Å, °) for (I)

Mn1—O5	2.079 (2)	C1—O2	1.256 (4)
Mn1—O2	2.102 (2)	C20—O4	1.254 (4)
Mn1—O3	2.171 (2)	C20—O5	1.257 (4)
Mn1—O6	2.188 (2)	C45—O8	1.251 (4)
Mn1—O7	2.282 (2)	C45—O7	1.253 (4)
Mn1—O8	2.306 (2)	C46—O10	1.241 (4)
Mn1—O13	2.580 (12)	C46—O9	1.256 (4)
C1—O1	1.251 (4)		
			
O5—Mn1—O2	94.69 (10)	O3—Mn1—O7	115.05 (9)
O5—Mn1—O3	156.29 (10)	O6—Mn1—O7	132.68 (9)
O2—Mn1—O3	81.86 (8)	O5—Mn1—O8	113.13 (10)
O5—Mn1—O6	82.05 (8)	O2—Mn1—O8	129.45 (10)
O2—Mn1—O6	141.70 (10)	O3—Mn1—O8	86.27 (8)
O3—Mn1—O6	86.26 (8)	O6—Mn1—O8	85.54 (8)
O5—Mn1—O7	87.81 (10)	O7—Mn1—O8	56.58 (8)
O2—Mn1—O7	84.96 (10)		

**Table 2:** Hydrogen-bond geometry (Å, °) for (I) *Cg*9 is the centroid of the C39–C44 ring.

*D*—H⋯*A*	*D*—H	H⋯*A*	*D*⋯*A*	*D*—H⋯*A*
N1—H1*A*⋯O8^i^	0.86	2.24	3.041 (3)	156
N1—H1*B*⋯O3	0.86	2.02	2.651 (3)	129
N4—H4*A*⋯O1^ii^	0.90	1.80	2.647 (4)	156
N4—H4*B*⋯O11^iii^	0.90	2.01	2.845 (3)	153
N5—H5*A*⋯O8^i^	0.86	2.13	2.954 (3)	160
N5—H5*B*⋯O6	0.86	2.01	2.651 (3)	131
N8—H8*A*⋯O4^iv^	0.90	1.85	2.750 (3)	175
N8—H8*B*⋯O11^v^	0.90	1.94	2.821 (3)	166
O11—H1*W*⋯O9^vi^	0.85	1.76	2.606 (3)	174
O11—H2*W*⋯O7^vii^	0.85	1.97	2.804 (3)	171
O12—H3*W*⋯O10^i^	0.85	2.11	2.924 (4)	159
O12—H4*W*⋯O10^vi^	0.85	2.00	2.844 (4)	174
O13—H5*W*⋯O2^vii^	0.95	2.41	3.000 (12)	120
C13—H13*B*⋯O4^vii^	0.97	2.56	3.526 (5)	178
C35—H35⋯O12^v^	0.98	2.38	3.321 (4)	161
C38—H38*A*⋯O13^v^	0.96	2.51	3.097 (12)	120
C12—H12*A*⋯*Cg*9^viii^	0.97	2.59	3.529 (4)	162

Symmetry codes: (i) 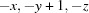; (ii) 
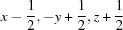
; (iii) 
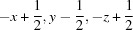
; (iv) 
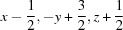
; (v) 
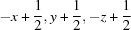
; (vi) 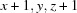; (vii) 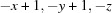; (viii) 
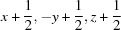
.

**Table 3:** Selected geometric parameters (Å, °) for (II)

Cu1—O2	1.889 (2)	C1—O2	1.283 (4)
Cu1—O3	1.9064 (18)	C23—O4	1.244 (4)
C1—O1	1.226 (4)	C23—O5	1.253 (4)
			
O2—Cu1—O3	93.24 (8)		

**Table 4:** Hydrogen-bond geometry (Å, °) for (II)

*D*—H⋯*A*	*D*—H	H⋯*A*	*D*⋯*A*	*D*—H⋯*A*
N1—H1*A*⋯O6^i^	0.86	2.19	2.995 (4)	155
N1—H1*B*⋯O3	0.86	1.98	2.604 (3)	129
N4—H4*A*⋯O5^ii^	0.90	1.80	2.658 (3)	160
N4—H4*B*⋯O4^iii^	0.90	1.90	2.777 (3)	166
O6—H1*W*⋯O1^iv^	0.84	1.95	2.716 (3)	151
O6—H2*W*⋯O1^v^	0.84	2.46	3.048 (4)	128
C12—H12*A*⋯O6^vi^	0.97	2.55	3.494 (5)	166
C13*B*—H13*D*⋯O1^ii^	0.97	2.52	3.113 (6)	119
C13*B*—H13*D*⋯O2^ii^	0.97	2.41	3.258 (6)	146

Symmetry codes: (i) 
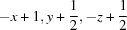
; (ii) 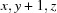; (iii) 
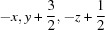
; (iv) 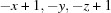; (v) 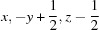; (vi) 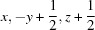.

**Table 5:** Experimental details

	(I)	(II)
Crystal data		
Chemical formula	[Mn(C_8_H_4_O_4_)(C_19_H_22_F_2_N_4_O_3_)_2_(H_2_O)_0.25_]·2H_2_O	[Cu(C_19_H_22_F_2_N_4_O_3_)_2_](C_8_H_4_O_4_)·2H_2_O
*M*_r_	1044.40	1048.50
Crystal system, space group	Monoclinic, *P*2_1_/*n*	Monoclinic, *P*2_1_/*c*
Temperature (K)	296	296
*a*, *b*, *c* (Å)	13.1128 (7), 20.8621 (12), 17.6284 (10)	13.6039 (2), 7.8019 (1), 22.0870 (3)
β (°)	106.725 (1)	103.764 (1)
*V* (Å^3^)	4618.4 (4)	2276.91 (5)
*Z*	4	2
Radiation type	Mo *K*α	Mo *K*α
μ (mm^−1^)	0.38	0.57
Crystal size (mm)	0.20 × 0.18 × 0.15	0.20 × 0.17 × 0.13

Data collection		
Diffractometer	Bruker SMART CCD	Bruker SMART CCD
Absorption correction	Multi-scan (*SADABS*; Bruker, 2004 ▸)	Multi-scan (*SADABS*; Bruker, 2004 ▸)
*T*_min_, *T*_max_	0.929, 0.946	0.895, 0.930
No. of measured, independent and observed [*I* > 2σ(*I*)] reflections	43573, 10603, 5832	20662, 5168, 3641
*R*_int_	0.082	0.049
(sin θ/λ)_max_ (Å^−1^)	0.650	0.647

Refinement		
*R*[*F* ^2^ > 2σ(*F* ^2^)], *wR*(*F* ^2^), *S*	0.059, 0.155, 1.04	0.052, 0.142, 1.06
No. of reflections	10603	5168
No. of parameters	648	323
H-atom treatment	H-atom parameters constrained	H-atom parameters constrained
Δρ_max_, Δρ_min_ (e Å^−3^)	0.59, −0.34	0.56, −0.57

Computer programs: *SMART* and *SAINT* (Bruker, 2004 ▸), *SHELXS97* and *SHELXL97* (Sheldrick, 2008 ▸), *ORTEP-3 for Windows* (Farrugia, 2012 ▸) and *publCIF* (Westrip, 2010 ▸).

## supplementary crystallographic information

### (I) 0.25-Aqua(benzene-1,4-dicarboxylato-κ2O,O')bis(sparfloxacin-κ2O,O')manganese(II) dihydrate Crystal data

**Table:**

[Mn(C_8_H_4_O_4_)(C_19_H_22_F_2_N_4_O_3_)_2_(H_2_O)_0.25_]·2H_2_O	*F*(000) = 2174
*M**_r_* = 1044.40	*D*_x_ = 1.502 Mg m^−^^3^
Monoclinic, *P*2_1_/*n*	Mo *K*α radiation, λ = 0.71073 Å
*a* = 13.1128 (7) Å	Cell parameters from 3519 reflections
*b* = 20.8621 (12) Å	θ = 2.4–20.8°
*c* = 17.6284 (10) Å	µ = 0.38 mm^−^^1^
β = 106.725 (1)°	*T* = 296 K
*V* = 4618.4 (4) Å^3^	Prism, colourless
*Z* = 4	0.20 × 0.18 × 0.15 mm

### (I) 0.25-Aqua(benzene-1,4-dicarboxylato-κ2O,O')bis(sparfloxacin-κ2O,O')manganese(II) dihydrate Data collection

**Table:**

Bruker SMART CCD diffractometer	10603 independent reflections
Radiation source: fine-focus sealed tube	5832 reflections with *I* > 2σ(*I*)
Graphite monochromator	*R*_int_ = 0.082
ω scans	θ_max_ = 27.5°, θ_min_ = 1.9°
Absorption correction: multi-scan (SADABS; Bruker, 2004)	*h* = −16→17
*T*_min_ = 0.929, *T*_max_ = 0.946	*k* = −27→26
43573 measured reflections	*l* = −22→22

### (I) 0.25-Aqua(benzene-1,4-dicarboxylato-κ2O,O')bis(sparfloxacin-κ2O,O')manganese(II) dihydrate Refinement

**Table:**

Refinement on *F*^2^	Primary atom site location: structure-invariant direct methods
Least-squares matrix: full	Secondary atom site location: difference Fourier map
*R*[*F*^2^ > 2σ(*F*^2^)] = 0.059	Hydrogen site location: inferred from neighbouring sites
*wR*(*F*^2^) = 0.155	H-atom parameters constrained
*S* = 1.04	*w* = 1/[σ^2^(*F*_o_^2^) + (0.0624*P*)^2^ + 0.3922*P*] where *P* = (*F*_o_^2^ + 2*F*_c_^2^)/3
10603 reflections	(Δ/σ)_max_ = 0.001
648 parameters	Δρ_max_ = 0.59 e Å^−^^3^
0 restraints	Δρ_min_ = −0.34 e Å^−^^3^

### (I) 0.25-Aqua(benzene-1,4-dicarboxylato-κ2O,O')bis(sparfloxacin-κ2O,O')manganese(II) dihydrate Special details

**Table:**

Geometry. All esds (except the esd in the dihedral angle between two l.s. planes) are estimated using the full covariance matrix. The cell esds are taken into account individually in the estimation of esds in distances, angles and torsion angles; correlations between esds in cell parameters are only used when they are defined by crystal symmetry. An approximate (isotropic) treatment of cell esds is used for estimating esds involving l.s. planes.
Refinement. Refinement of F^2^ against ALL reflections. The weighted R-factor wR and goodness of fit S are based on F^2^, conventional R-factors R are based on F, with F set to zero for negative F^2^. The threshold expression of F^2^ > 2sigma(F^2^) is used only for calculating R-factors(gt) etc. and is not relevant to the choice of reflections for refinement. R-factors based on F^2^ are statistically about twice as large as those based on F, and R- factors based on ALL data will be even larger.

### (I) 0.25-Aqua(benzene-1,4-dicarboxylato-κ2O,O')bis(sparfloxacin-κ2O,O')manganese(II) dihydrate Fractional atomic coordinates and isotropic or equivalent isotropic displacement parameters (Å^2^)

**Table:**

	*x*	*y*	*z*	*U*_iso_*/*U*_eq_	Occ. (<1)
Mn1	0.24557 (4)	0.50546 (2)	−0.08355 (3)	0.03353 (14)	
C1	0.3662 (3)	0.37777 (17)	−0.0926 (2)	0.0436 (8)	
C2	0.3073 (2)	0.34168 (14)	−0.04545 (17)	0.0343 (7)	
C3	0.2279 (2)	0.36801 (15)	−0.01256 (16)	0.0328 (7)	
C4	0.1766 (2)	0.32345 (14)	0.02819 (17)	0.0341 (7)	
C5	0.0966 (2)	0.34353 (16)	0.06350 (18)	0.0388 (8)	
C6	0.0445 (3)	0.29665 (18)	0.0930 (2)	0.0488 (9)	
C7	0.0655 (3)	0.23150 (18)	0.0926 (2)	0.0530 (10)	
C8	0.1433 (3)	0.21331 (16)	0.0586 (2)	0.0497 (9)	
C9	0.2041 (2)	0.25707 (15)	0.03201 (18)	0.0380 (8)	
C10	0.3369 (2)	0.27935 (15)	−0.02990 (18)	0.0391 (8)	
H10	0.3935	0.2647	−0.0470	0.047*	
C11	0.3414 (3)	0.17419 (15)	0.0295 (2)	0.0467 (9)	
H11	0.3110	0.1386	−0.0061	0.056*	
C12	0.3858 (3)	0.15806 (17)	0.1143 (2)	0.0547 (10)	
H12A	0.3813	0.1138	0.1299	0.066*	
H12B	0.3788	0.1896	0.1529	0.066*	
C13	0.4591 (3)	0.17312 (19)	0.0659 (2)	0.0644 (11)	
H13A	0.4988	0.1380	0.0520	0.077*	
H13B	0.4963	0.2139	0.0750	0.077*	
C14	−0.0986 (3)	0.1819 (2)	0.1131 (2)	0.0652 (11)	
H14A	−0.1236	0.1457	0.0781	0.078*	
H14B	−0.1331	0.2202	0.0867	0.078*	
C15	−0.1268 (3)	0.1725 (2)	0.1865 (2)	0.0589 (10)	
H15	−0.1085	0.2120	0.2175	0.071*	
C16	0.0533 (3)	0.12232 (19)	0.2441 (2)	0.0574 (10)	
H16	0.0815	0.1602	0.2763	0.069*	
C17	0.0725 (3)	0.1306 (2)	0.1677 (3)	0.0833 (15)	
H17A	0.0476	0.0932	0.1349	0.100*	
H17B	0.1483	0.1353	0.1747	0.100*	
C18	−0.2450 (3)	0.1593 (2)	0.1752 (3)	0.0715 (12)	
H18A	−0.2859	0.1964	0.1527	0.107*	
H18B	−0.2560	0.1499	0.2256	0.107*	
H18C	−0.2673	0.1234	0.1403	0.107*	
C19	0.1081 (3)	0.06386 (17)	0.2888 (2)	0.0610 (11)	
H19A	0.0833	0.0261	0.2579	0.091*	
H19B	0.0922	0.0607	0.3385	0.091*	
H19C	0.1837	0.0678	0.2982	0.091*	
N1	0.0748 (2)	0.40641 (14)	0.07178 (16)	0.0518 (8)	
H1A	0.0284	0.4170	0.0955	0.062*	
H1B	0.1074	0.4356	0.0533	0.062*	
N2	0.2918 (2)	0.23737 (12)	0.00780 (15)	0.0383 (6)	
N3	0.0158 (2)	0.18803 (16)	0.1292 (2)	0.0799 (12)	
N4	−0.0632 (2)	0.11872 (13)	0.23537 (16)	0.0450 (7)	
H4A	−0.0731	0.1195	0.2838	0.054*	
H4B	−0.0884	0.0811	0.2126	0.054*	
O1	0.4253 (2)	0.34607 (12)	−0.12306 (16)	0.0638 (7)	
O2	0.3552 (2)	0.43743 (11)	−0.10042 (16)	0.0626 (7)	
O3	0.20213 (16)	0.42675 (10)	−0.01847 (12)	0.0388 (5)	
F1	−0.02880 (15)	0.31754 (10)	0.12892 (12)	0.0642 (6)	
F2	0.15513 (17)	0.14946 (10)	0.04660 (15)	0.0718 (7)	
C20	0.3488 (3)	0.64075 (16)	−0.07543 (19)	0.0410 (8)	
C21	0.3025 (2)	0.66361 (14)	−0.01229 (17)	0.0327 (7)	
C22	0.2345 (2)	0.62688 (14)	0.02221 (16)	0.0304 (7)	
C23	0.1873 (2)	0.66087 (14)	0.07591 (16)	0.0306 (7)	
C24	0.1099 (2)	0.63154 (14)	0.10809 (17)	0.0330 (7)	
C25	0.0609 (2)	0.66937 (16)	0.15148 (19)	0.0391 (8)	
C26	0.0803 (2)	0.73363 (16)	0.1662 (2)	0.0418 (8)	
C27	0.1566 (3)	0.76117 (15)	0.1359 (2)	0.0421 (8)	
C28	0.2130 (2)	0.72662 (15)	0.09484 (18)	0.0340 (7)	
C29	0.3311 (2)	0.72359 (15)	0.01592 (18)	0.0366 (7)	
H29	0.3808	0.7449	−0.0033	0.044*	
C30	0.3460 (3)	0.81431 (15)	0.1034 (2)	0.0458 (9)	
H30	0.3127	0.8543	0.0793	0.055*	
C31	0.3993 (3)	0.81582 (18)	0.1898 (2)	0.0588 (10)	
H31A	0.3964	0.7774	0.2201	0.071*	
H31B	0.3976	0.8557	0.2177	0.071*	
C32	0.4648 (3)	0.81376 (19)	0.1328 (3)	0.0674 (12)	
H32A	0.5023	0.8524	0.1261	0.081*	
H32B	0.5012	0.7740	0.1285	0.081*	
C33	−0.0843 (2)	0.76537 (17)	0.2025 (2)	0.0481 (9)	
H33A	−0.0922	0.7443	0.2495	0.058*	
H33B	−0.1194	0.7394	0.1568	0.058*	
C34	−0.1347 (2)	0.83148 (17)	0.19441 (19)	0.0437 (8)	
H34	−0.1318	0.8506	0.1443	0.052*	
C35	0.0423 (2)	0.87778 (15)	0.27129 (18)	0.0380 (8)	
H35	0.0548	0.9011	0.2266	0.046*	
C36	0.0863 (2)	0.81119 (15)	0.2745 (2)	0.0439 (8)	
H36A	0.1603	0.8135	0.2747	0.053*	
H36B	0.0840	0.7909	0.3235	0.053*	
C37	−0.2504 (3)	0.8294 (2)	0.1959 (2)	0.0652 (11)	
H37A	−0.2536	0.8123	0.2457	0.098*	
H37B	−0.2908	0.8025	0.1537	0.098*	
H37C	−0.2794	0.8719	0.1892	0.098*	
C38	0.0920 (3)	0.91361 (16)	0.3473 (2)	0.0535 (10)	
H38A	0.0518	0.9518	0.3487	0.080*	
H38B	0.1639	0.9250	0.3500	0.080*	
H38C	0.0921	0.8868	0.3916	0.080*	
N5	0.0885 (2)	0.56800 (12)	0.10051 (15)	0.0446 (7)	
H5A	0.0444	0.5513	0.1227	0.053*	
H5B	0.1189	0.5444	0.0735	0.053*	
N6	0.2939 (2)	0.75471 (11)	0.06912 (15)	0.0360 (6)	
N7	0.0278 (2)	0.77193 (14)	0.20811 (18)	0.0532 (8)	
N8	−0.07495 (19)	0.87282 (12)	0.26085 (14)	0.0377 (6)	
H8A	−0.0846	0.8576	0.3060	0.045*	
H8B	−0.1030	0.9125	0.2533	0.045*	
O4	0.4009 (2)	0.68048 (11)	−0.10270 (14)	0.0559 (7)	
O5	0.3336 (2)	0.58419 (12)	−0.10073 (15)	0.0627 (8)	
O6	0.21452 (16)	0.56810 (10)	0.00687 (12)	0.0382 (5)	
F3	−0.00977 (15)	0.63937 (9)	0.18330 (11)	0.0524 (5)	
F4	0.16868 (16)	0.82596 (9)	0.14286 (13)	0.0595 (6)	
C39	−0.0089 (2)	0.50896 (15)	−0.28773 (18)	0.0386 (8)	
C40	−0.1129 (3)	0.51354 (16)	−0.28443 (19)	0.0417 (8)	
H40	−0.1266	0.5163	−0.2356	0.050*	
C41	−0.1973 (3)	0.51408 (16)	−0.35408 (19)	0.0433 (8)	
H41	−0.2669	0.5175	−0.3513	0.052*	
C42	−0.1783 (3)	0.50961 (15)	−0.42676 (18)	0.0396 (8)	
C43	−0.0744 (3)	0.50486 (19)	−0.4288 (2)	0.0557 (10)	
H43	−0.0608	0.5012	−0.4776	0.067*	
C44	0.0102 (3)	0.50530 (18)	−0.3607 (2)	0.0544 (10)	
H44	0.0797	0.5032	−0.3640	0.065*	
C45	0.0824 (3)	0.50855 (15)	−0.21292 (19)	0.0383 (8)	
C46	−0.2660 (3)	0.50852 (18)	−0.5041 (2)	0.0501 (9)	
O7	0.17498 (19)	0.50044 (13)	−0.21779 (14)	0.0646 (8)	
O8	0.06601 (16)	0.51510 (10)	−0.14685 (12)	0.0407 (5)	
O9	−0.2441 (2)	0.48093 (17)	−0.56076 (15)	0.0837 (10)	
O10	−0.3529 (2)	0.53337 (14)	−0.50676 (15)	0.0693 (8)	
O11	0.65934 (16)	0.49527 (10)	0.28884 (12)	0.0432 (6)	
H1W	0.6939	0.4892	0.3369	0.052*	
H2W	0.7131	0.4987	0.2717	0.052*	
O12	0.4787 (2)	0.45928 (17)	0.39436 (16)	0.0930 (10)	
H3W	0.4443	0.4509	0.4274	0.112*	
H4W	0.5267	0.4809	0.4268	0.112*	
O13	0.4286 (9)	0.5077 (5)	0.0220 (7)	0.076 (3)*	0.25
H5W	0.4567	0.5407	0.0593	0.091*	0.25
H6W	0.5000	0.5000	0.0000	0.091*	0.50

### (I) 0.25-Aqua(benzene-1,4-dicarboxylato-κ2O,O')bis(sparfloxacin-κ2O,O')manganese(II) dihydrate Atomic displacement parameters (Å^2^)

**Table:**

	*U*^11^	*U*^22^	*U*^33^	*U*^12^	*U*^13^	*U*^23^
Mn1	0.0424 (3)	0.0305 (3)	0.0320 (2)	−0.0020 (2)	0.0176 (2)	−0.0009 (2)
C1	0.046 (2)	0.041 (2)	0.049 (2)	0.0027 (16)	0.0222 (16)	0.0071 (17)
C2	0.0387 (18)	0.0330 (18)	0.0333 (17)	−0.0017 (14)	0.0137 (14)	0.0015 (13)
C3	0.0371 (17)	0.0343 (18)	0.0263 (15)	−0.0020 (14)	0.0081 (13)	0.0003 (13)
C4	0.0339 (17)	0.0375 (18)	0.0317 (16)	0.0003 (14)	0.0106 (13)	0.0061 (13)
C5	0.0384 (19)	0.042 (2)	0.0365 (18)	0.0061 (15)	0.0120 (14)	0.0119 (15)
C6	0.039 (2)	0.065 (3)	0.049 (2)	0.0115 (18)	0.0243 (16)	0.0196 (18)
C7	0.040 (2)	0.058 (2)	0.066 (2)	0.0037 (18)	0.0230 (18)	0.025 (2)
C8	0.047 (2)	0.036 (2)	0.069 (3)	0.0022 (16)	0.0215 (19)	0.0094 (18)
C9	0.0375 (18)	0.0371 (19)	0.0399 (18)	−0.0018 (15)	0.0119 (15)	0.0057 (14)
C10	0.0423 (19)	0.041 (2)	0.0382 (18)	−0.0003 (15)	0.0189 (15)	0.0016 (15)
C11	0.054 (2)	0.0290 (19)	0.060 (2)	0.0003 (16)	0.0196 (18)	0.0022 (16)
C12	0.068 (3)	0.038 (2)	0.057 (2)	0.0013 (18)	0.017 (2)	0.0057 (17)
C13	0.053 (2)	0.049 (2)	0.097 (3)	0.0092 (19)	0.029 (2)	0.016 (2)
C14	0.049 (2)	0.069 (3)	0.084 (3)	0.006 (2)	0.029 (2)	0.027 (2)
C15	0.046 (2)	0.065 (3)	0.074 (3)	0.0120 (19)	0.032 (2)	0.018 (2)
C16	0.047 (2)	0.058 (3)	0.071 (3)	0.0030 (18)	0.0230 (19)	0.018 (2)
C17	0.059 (3)	0.086 (3)	0.118 (4)	0.025 (2)	0.046 (3)	0.062 (3)
C18	0.048 (2)	0.081 (3)	0.095 (3)	0.006 (2)	0.035 (2)	0.024 (3)
C19	0.066 (3)	0.047 (2)	0.070 (3)	0.0023 (19)	0.019 (2)	0.0178 (19)
N1	0.0566 (19)	0.0507 (19)	0.0611 (19)	0.0077 (15)	0.0377 (15)	0.0062 (15)
N2	0.0419 (16)	0.0286 (15)	0.0485 (16)	0.0008 (12)	0.0195 (13)	0.0028 (12)
N3	0.0408 (19)	0.079 (3)	0.131 (3)	0.0176 (17)	0.0425 (19)	0.071 (2)
N4	0.0458 (17)	0.0467 (18)	0.0501 (17)	−0.0003 (14)	0.0260 (13)	0.0048 (14)
O1	0.0841 (19)	0.0505 (16)	0.0784 (19)	0.0172 (14)	0.0578 (16)	0.0129 (13)
O2	0.0676 (17)	0.0416 (16)	0.100 (2)	0.0070 (13)	0.0576 (16)	0.0205 (14)
O3	0.0457 (13)	0.0321 (13)	0.0436 (13)	0.0036 (10)	0.0209 (10)	0.0044 (10)
F1	0.0519 (13)	0.0850 (16)	0.0689 (14)	0.0212 (11)	0.0385 (11)	0.0319 (12)
F2	0.0669 (15)	0.0401 (13)	0.118 (2)	−0.0066 (11)	0.0416 (13)	0.0121 (12)
C20	0.052 (2)	0.040 (2)	0.0397 (18)	−0.0097 (16)	0.0279 (16)	−0.0038 (15)
C21	0.0347 (17)	0.0329 (17)	0.0338 (16)	−0.0065 (14)	0.0154 (13)	−0.0029 (13)
C22	0.0345 (17)	0.0328 (18)	0.0255 (15)	−0.0014 (13)	0.0111 (12)	−0.0015 (13)
C23	0.0295 (16)	0.0346 (17)	0.0296 (15)	−0.0033 (13)	0.0114 (13)	−0.0007 (13)
C24	0.0351 (17)	0.0347 (18)	0.0317 (16)	−0.0057 (14)	0.0137 (13)	−0.0023 (13)
C25	0.0353 (18)	0.046 (2)	0.0446 (19)	−0.0070 (15)	0.0252 (15)	−0.0051 (15)
C26	0.0342 (18)	0.047 (2)	0.051 (2)	0.0016 (15)	0.0229 (15)	−0.0113 (16)
C27	0.0406 (19)	0.0324 (19)	0.059 (2)	−0.0032 (15)	0.0237 (17)	−0.0113 (16)
C28	0.0299 (16)	0.0356 (18)	0.0397 (17)	−0.0036 (14)	0.0151 (14)	−0.0019 (14)
C29	0.0374 (18)	0.0364 (18)	0.0421 (18)	−0.0039 (15)	0.0214 (14)	0.0006 (15)
C30	0.051 (2)	0.0261 (18)	0.067 (2)	−0.0064 (15)	0.0277 (18)	−0.0099 (16)
C31	0.065 (3)	0.048 (2)	0.063 (3)	−0.0039 (19)	0.017 (2)	−0.0184 (19)
C32	0.048 (2)	0.056 (3)	0.105 (3)	−0.0159 (19)	0.033 (2)	−0.031 (2)
C33	0.0345 (19)	0.055 (2)	0.060 (2)	−0.0084 (16)	0.0215 (16)	−0.0211 (18)
C34	0.0398 (19)	0.057 (2)	0.0363 (18)	−0.0026 (16)	0.0144 (15)	−0.0093 (16)
C35	0.0423 (19)	0.0405 (19)	0.0336 (17)	−0.0063 (15)	0.0146 (14)	0.0005 (14)
C36	0.0354 (19)	0.048 (2)	0.052 (2)	−0.0035 (16)	0.0185 (16)	−0.0131 (17)
C37	0.042 (2)	0.077 (3)	0.080 (3)	−0.004 (2)	0.0228 (19)	−0.028 (2)
C38	0.072 (3)	0.037 (2)	0.045 (2)	−0.0032 (18)	0.0061 (18)	0.0001 (16)
N5	0.0535 (17)	0.0410 (17)	0.0513 (17)	−0.0104 (13)	0.0345 (14)	−0.0059 (13)
N6	0.0394 (15)	0.0280 (14)	0.0470 (16)	−0.0041 (11)	0.0226 (12)	−0.0065 (12)
N7	0.0319 (15)	0.064 (2)	0.071 (2)	−0.0095 (14)	0.0270 (14)	−0.0376 (17)
N8	0.0455 (16)	0.0370 (15)	0.0344 (14)	0.0041 (12)	0.0177 (12)	0.0004 (12)
O4	0.0775 (18)	0.0497 (15)	0.0598 (16)	−0.0206 (13)	0.0504 (14)	−0.0114 (12)
O5	0.091 (2)	0.0476 (16)	0.0744 (18)	−0.0278 (14)	0.0633 (16)	−0.0244 (13)
O6	0.0501 (13)	0.0321 (13)	0.0400 (12)	−0.0090 (10)	0.0251 (10)	−0.0071 (10)
F3	0.0562 (12)	0.0552 (13)	0.0614 (13)	−0.0126 (10)	0.0417 (10)	−0.0118 (10)
F4	0.0617 (13)	0.0364 (12)	0.0949 (16)	−0.0015 (10)	0.0457 (12)	−0.0127 (11)
C39	0.0347 (18)	0.044 (2)	0.0374 (17)	−0.0039 (15)	0.0105 (14)	0.0026 (15)
C40	0.0397 (19)	0.053 (2)	0.0344 (17)	0.0005 (16)	0.0143 (14)	0.0001 (15)
C41	0.0341 (18)	0.055 (2)	0.0433 (19)	−0.0001 (15)	0.0145 (15)	−0.0023 (16)
C42	0.0400 (19)	0.043 (2)	0.0354 (17)	−0.0008 (15)	0.0105 (14)	0.0024 (14)
C43	0.048 (2)	0.094 (3)	0.0303 (18)	−0.006 (2)	0.0192 (15)	−0.0058 (18)
C44	0.0374 (19)	0.088 (3)	0.041 (2)	−0.0055 (19)	0.0171 (16)	0.0001 (19)
C45	0.0412 (19)	0.0384 (19)	0.0376 (18)	−0.0061 (15)	0.0152 (15)	−0.0006 (14)
C46	0.049 (2)	0.063 (3)	0.0366 (19)	−0.0037 (19)	0.0100 (16)	0.0060 (17)
O7	0.0348 (14)	0.119 (2)	0.0441 (14)	0.0013 (14)	0.0173 (11)	−0.0041 (14)
O8	0.0404 (13)	0.0515 (14)	0.0339 (12)	−0.0040 (10)	0.0163 (10)	−0.0003 (10)
O9	0.0639 (19)	0.145 (3)	0.0373 (15)	0.0127 (18)	0.0075 (13)	−0.0132 (17)
O10	0.0480 (17)	0.096 (2)	0.0560 (16)	0.0152 (16)	0.0026 (13)	−0.0057 (15)
O11	0.0371 (12)	0.0573 (15)	0.0366 (12)	−0.0025 (11)	0.0128 (10)	0.0039 (10)
O12	0.076 (2)	0.146 (3)	0.0573 (18)	−0.027 (2)	0.0191 (15)	−0.0331 (19)

### (I) 0.25-Aqua(benzene-1,4-dicarboxylato-κ2O,O')bis(sparfloxacin-κ2O,O')manganese(II) dihydrate Geometric parameters (Å, º)

**Table:**

Mn1—O5	2.079 (2)	C23—C24	1.434 (4)
Mn1—O2	2.102 (2)	C24—N5	1.354 (4)
Mn1—O3	2.171 (2)	C24—C25	1.380 (4)
Mn1—O6	2.188 (2)	C25—F3	1.365 (3)
Mn1—O7	2.282 (2)	C25—C26	1.375 (4)
Mn1—O8	2.306 (2)	C26—C27	1.386 (4)
Mn1—O13	2.580 (12)	C26—N7	1.398 (4)
C1—O1	1.251 (4)	C27—F4	1.362 (3)
C1—O2	1.256 (4)	C27—C28	1.378 (4)
C1—C2	1.491 (4)	C28—N6	1.397 (4)
C2—C10	1.362 (4)	C29—N6	1.343 (4)
C2—C3	1.439 (4)	C29—H29	0.9300
C3—O3	1.267 (3)	C30—N6	1.463 (4)
C3—C4	1.452 (4)	C30—C31	1.482 (5)
C4—C9	1.428 (4)	C30—C32	1.493 (5)
C4—C5	1.429 (4)	C30—H30	0.9800
C5—N1	1.359 (4)	C31—C32	1.498 (5)
C5—C6	1.377 (4)	C31—H31A	0.9700
C6—F1	1.367 (4)	C31—H31B	0.9700
C6—C7	1.387 (5)	C32—H32A	0.9700
C7—C8	1.377 (5)	C32—H32B	0.9700
C7—N3	1.381 (4)	C33—N7	1.450 (4)
C8—F2	1.364 (4)	C33—C34	1.519 (5)
C8—C9	1.380 (4)	C33—H33A	0.9700
C9—N2	1.398 (4)	C33—H33B	0.9700
C10—N2	1.336 (4)	C34—N8	1.484 (4)
C10—H10	0.9300	C34—C37	1.524 (4)
C11—N2	1.471 (4)	C34—H34	0.9800
C11—C12	1.478 (5)	C35—N8	1.499 (4)
C11—C13	1.491 (5)	C35—C36	1.499 (4)
C11—H11	0.9800	C35—C38	1.509 (4)
C12—C13	1.491 (5)	C35—H35	0.9800
C12—H12A	0.9700	C36—N7	1.453 (4)
C12—H12B	0.9700	C36—H36A	0.9700
C13—H13A	0.9700	C36—H36B	0.9700
C13—H13B	0.9700	C37—H37A	0.9600
C14—N3	1.450 (4)	C37—H37B	0.9600
C14—C15	1.457 (5)	C37—H37C	0.9600
C14—H14A	0.9700	C38—H38A	0.9600
C14—H14B	0.9700	C38—H38B	0.9600
C15—N4	1.511 (4)	C38—H38C	0.9600
C15—C18	1.530 (5)	N5—H5A	0.8600
C15—H15	0.9800	N5—H5B	0.8600
C16—C17	1.450 (5)	N8—H8A	0.9000
C16—N4	1.493 (4)	N8—H8B	0.9000
C16—C19	1.516 (5)	C39—C44	1.382 (4)
C16—H16	0.9800	C39—C40	1.385 (4)
C17—N3	1.470 (4)	C39—C45	1.505 (4)
C17—H17A	0.9700	C40—C41	1.397 (4)
C17—H17B	0.9700	C40—H40	0.9300
C18—H18A	0.9600	C41—C42	1.377 (4)
C18—H18B	0.9600	C41—H41	0.9300
C18—H18C	0.9600	C42—C43	1.376 (5)
C19—H19A	0.9600	C42—C46	1.510 (4)
C19—H19B	0.9600	C43—C44	1.380 (5)
C19—H19C	0.9600	C43—H43	0.9300
N1—H1A	0.8600	C44—H44	0.9300
N1—H1B	0.8600	C45—O8	1.251 (4)
N4—H4A	0.9000	C45—O7	1.253 (4)
N4—H4B	0.9000	C46—O10	1.241 (4)
C20—O4	1.254 (4)	C46—O9	1.256 (4)
C20—O5	1.257 (4)	O11—H1W	0.8467
C20—C21	1.492 (4)	O11—H2W	0.8454
C21—C29	1.359 (4)	O12—H3W	0.8505
C21—C22	1.436 (4)	O12—H4W	0.8491
C22—O6	1.267 (3)	O13—H5W	0.9503
C22—C23	1.457 (4)	O13—H6W	1.1238
C23—C28	1.429 (4)		
			
O5—Mn1—O2	94.69 (10)	O6—C22—C23	120.6 (3)
O5—Mn1—O3	156.29 (10)	C21—C22—C23	116.6 (3)
O2—Mn1—O3	81.86 (8)	C28—C23—C24	117.6 (3)
O5—Mn1—O6	82.05 (8)	C28—C23—C22	120.2 (3)
O2—Mn1—O6	141.70 (10)	C24—C23—C22	122.1 (3)
O3—Mn1—O6	86.26 (8)	N5—C24—C25	119.9 (3)
O5—Mn1—O7	87.81 (10)	N5—C24—C23	121.9 (3)
O2—Mn1—O7	84.96 (10)	C25—C24—C23	118.1 (3)
O3—Mn1—O7	115.05 (9)	F3—C25—C26	118.7 (3)
O6—Mn1—O7	132.68 (9)	F3—C25—C24	116.4 (3)
O5—Mn1—O8	113.13 (10)	C26—C25—C24	124.8 (3)
O2—Mn1—O8	129.45 (10)	C25—C26—C27	116.5 (3)
O3—Mn1—O8	86.27 (8)	C25—C26—N7	124.3 (3)
O6—Mn1—O8	85.54 (8)	C27—C26—N7	119.1 (3)
O7—Mn1—O8	56.58 (8)	F4—C27—C28	120.0 (3)
O1—C1—O2	122.8 (3)	F4—C27—C26	117.0 (3)
O1—C1—C2	117.1 (3)	C28—C27—C26	122.8 (3)
O2—C1—C2	120.0 (3)	C27—C28—N6	121.6 (3)
C10—C2—C3	118.7 (3)	C27—C28—C23	119.9 (3)
C10—C2—C1	115.6 (3)	N6—C28—C23	118.5 (3)
C3—C2—C1	125.6 (3)	N6—C29—C21	125.7 (3)
O3—C3—C2	122.9 (3)	N6—C29—H29	117.2
O3—C3—C4	120.6 (3)	C21—C29—H29	117.2
C2—C3—C4	116.5 (3)	N6—C30—C31	118.0 (3)
C9—C4—C5	118.1 (3)	N6—C30—C32	117.0 (3)
C9—C4—C3	119.9 (3)	C31—C30—C32	60.5 (3)
C5—C4—C3	122.0 (3)	N6—C30—H30	116.6
N1—C5—C6	120.2 (3)	C31—C30—H30	116.6
N1—C5—C4	122.2 (3)	C32—C30—H30	116.6
C6—C5—C4	117.5 (3)	C30—C31—C32	60.1 (2)
F1—C6—C5	116.1 (3)	C30—C31—H31A	117.8
F1—C6—C7	118.8 (3)	C32—C31—H31A	117.8
C5—C6—C7	125.0 (3)	C30—C31—H31B	117.8
C8—C7—N3	122.1 (3)	C32—C31—H31B	117.8
C8—C7—C6	116.4 (3)	H31A—C31—H31B	114.9
N3—C7—C6	121.4 (3)	C30—C32—C31	59.4 (2)
F2—C8—C7	117.9 (3)	C30—C32—H32A	117.8
F2—C8—C9	119.4 (3)	C31—C32—H32A	117.8
C7—C8—C9	122.6 (3)	C30—C32—H32B	117.8
C8—C9—N2	121.0 (3)	C31—C32—H32B	117.8
C8—C9—C4	119.7 (3)	H32A—C32—H32B	115.0
N2—C9—C4	119.3 (3)	N7—C33—C34	109.1 (3)
N2—C10—C2	125.8 (3)	N7—C33—H33A	109.9
N2—C10—H10	117.1	C34—C33—H33A	109.9
C2—C10—H10	117.1	N7—C33—H33B	109.9
N2—C11—C12	118.9 (3)	C34—C33—H33B	109.9
N2—C11—C13	116.8 (3)	H33A—C33—H33B	108.3
C12—C11—C13	60.3 (2)	N8—C34—C33	109.7 (3)
N2—C11—H11	116.4	N8—C34—C37	107.8 (3)
C12—C11—H11	116.4	C33—C34—C37	112.4 (3)
C13—C11—H11	116.4	N8—C34—H34	109.0
C11—C12—C13	60.3 (2)	C33—C34—H34	109.0
C11—C12—H12A	117.7	C37—C34—H34	109.0
C13—C12—H12A	117.7	N8—C35—C36	108.1 (2)
C11—C12—H12B	117.7	N8—C35—C38	108.0 (3)
C13—C12—H12B	117.7	C36—C35—C38	111.2 (3)
H12A—C12—H12B	114.9	N8—C35—H35	109.8
C12—C13—C11	59.4 (2)	C36—C35—H35	109.8
C12—C13—H13A	117.8	C38—C35—H35	109.8
C11—C13—H13A	117.8	N7—C36—C35	112.6 (3)
C12—C13—H13B	117.8	N7—C36—H36A	109.1
C11—C13—H13B	117.8	C35—C36—H36A	109.1
H13A—C13—H13B	115.0	N7—C36—H36B	109.1
N3—C14—C15	110.5 (3)	C35—C36—H36B	109.1
N3—C14—H14A	109.6	H36A—C36—H36B	107.8
C15—C14—H14A	109.6	C34—C37—H37A	109.5
N3—C14—H14B	109.6	C34—C37—H37B	109.5
C15—C14—H14B	109.6	H37A—C37—H37B	109.5
H14A—C14—H14B	108.1	C34—C37—H37C	109.5
C14—C15—N4	111.6 (3)	H37A—C37—H37C	109.5
C14—C15—C18	114.5 (3)	H37B—C37—H37C	109.5
N4—C15—C18	108.2 (3)	C35—C38—H38A	109.5
C14—C15—H15	107.4	C35—C38—H38B	109.5
N4—C15—H15	107.4	H38A—C38—H38B	109.5
C18—C15—H15	107.4	C35—C38—H38C	109.5
C17—C16—N4	110.9 (3)	H38A—C38—H38C	109.5
C17—C16—C19	113.2 (3)	H38B—C38—H38C	109.5
N4—C16—C19	109.4 (3)	C24—N5—H5A	120.0
C17—C16—H16	107.7	C24—N5—H5B	120.0
N4—C16—H16	107.7	H5A—N5—H5B	120.0
C19—C16—H16	107.7	C29—N6—C28	119.1 (3)
C16—C17—N3	109.0 (4)	C29—N6—C30	118.6 (3)
C16—C17—H17A	109.9	C28—N6—C30	122.0 (3)
N3—C17—H17A	109.9	C26—N7—C33	123.6 (3)
C16—C17—H17B	109.9	C26—N7—C36	121.4 (3)
N3—C17—H17B	109.9	C33—N7—C36	113.5 (2)
H17A—C17—H17B	108.3	C34—N8—C35	115.3 (2)
C15—C18—H18A	109.5	C34—N8—H8A	108.4
C15—C18—H18B	109.5	C35—N8—H8A	108.4
H18A—C18—H18B	109.5	C34—N8—H8B	108.4
C15—C18—H18C	109.5	C35—N8—H8B	108.4
H18A—C18—H18C	109.5	H8A—N8—H8B	107.5
H18B—C18—H18C	109.5	C20—O5—Mn1	136.7 (2)
C16—C19—H19A	109.5	C22—O6—Mn1	131.53 (18)
C16—C19—H19B	109.5	C44—C39—C40	119.2 (3)
H19A—C19—H19B	109.5	C44—C39—C45	120.3 (3)
C16—C19—H19C	109.5	C40—C39—C45	120.6 (3)
H19A—C19—H19C	109.5	C39—C40—C41	120.3 (3)
H19B—C19—H19C	109.5	C39—C40—H40	119.9
C5—N1—H1A	120.0	C41—C40—H40	119.9
C5—N1—H1B	120.0	C42—C41—C40	120.5 (3)
H1A—N1—H1B	120.0	C42—C41—H41	119.8
C10—N2—C9	118.7 (3)	C40—C41—H41	119.8
C10—N2—C11	119.4 (3)	C43—C42—C41	118.3 (3)
C9—N2—C11	121.7 (3)	C43—C42—C46	118.5 (3)
C7—N3—C14	124.6 (3)	C41—C42—C46	123.1 (3)
C7—N3—C17	120.4 (3)	C42—C43—C44	122.1 (3)
C14—N3—C17	112.1 (3)	C42—C43—H43	119.0
C16—N4—C15	113.7 (3)	C44—C43—H43	119.0
C16—N4—H4A	108.8	C43—C44—C39	119.6 (3)
C15—N4—H4A	108.8	C43—C44—H44	120.2
C16—N4—H4B	108.8	C39—C44—H44	120.2
C15—N4—H4B	108.8	O8—C45—O7	120.5 (3)
H4A—N4—H4B	107.7	O8—C45—C39	120.6 (3)
C1—O2—Mn1	135.6 (2)	O7—C45—C39	118.9 (3)
C3—O3—Mn1	132.10 (19)	O10—C46—O9	125.4 (3)
O4—C20—O5	122.2 (3)	O10—C46—C42	118.7 (3)
O4—C20—C21	117.3 (3)	O9—C46—C42	115.9 (3)
O5—C20—C21	120.5 (3)	C45—O7—Mn1	91.98 (19)
C29—C21—C22	118.4 (3)	C45—O8—Mn1	90.91 (19)
C29—C21—C20	116.0 (3)	H1W—O11—H2W	96.2
C22—C21—C20	125.5 (3)	H3W—O12—H4W	94.9
O6—C22—C21	122.8 (3)	H5W—O13—H6W	98.4
			
O1—C1—C2—C10	10.0 (5)	N5—C24—C25—F3	−1.4 (4)
O2—C1—C2—C10	−170.0 (3)	C23—C24—C25—F3	−178.0 (3)
O1—C1—C2—C3	−173.4 (3)	N5—C24—C25—C26	176.8 (3)
O2—C1—C2—C3	6.6 (5)	C23—C24—C25—C26	0.3 (5)
C10—C2—C3—O3	174.0 (3)	F3—C25—C26—C27	176.9 (3)
C1—C2—C3—O3	−2.6 (5)	C24—C25—C26—C27	−1.3 (5)
C10—C2—C3—C4	−6.4 (4)	F3—C25—C26—N7	−3.9 (5)
C1—C2—C3—C4	177.0 (3)	C24—C25—C26—N7	177.9 (3)
O3—C3—C4—C9	177.9 (3)	C25—C26—C27—F4	173.4 (3)
C2—C3—C4—C9	−1.7 (4)	N7—C26—C27—F4	−5.9 (5)
O3—C3—C4—C5	−0.5 (4)	C25—C26—C27—C28	−1.7 (5)
C2—C3—C4—C5	179.9 (3)	N7—C26—C27—C28	179.1 (3)
C9—C4—C5—N1	171.4 (3)	F4—C27—C28—N6	9.1 (5)
C3—C4—C5—N1	−10.2 (5)	C26—C27—C28—N6	−176.0 (3)
C9—C4—C5—C6	−5.5 (4)	F4—C27—C28—C23	−169.3 (3)
C3—C4—C5—C6	172.9 (3)	C26—C27—C28—C23	5.6 (5)
N1—C5—C6—F1	0.7 (5)	C24—C23—C28—C27	−6.3 (4)
C4—C5—C6—F1	177.7 (3)	C22—C23—C28—C27	170.3 (3)
N1—C5—C6—C7	−175.4 (3)	C24—C23—C28—N6	175.2 (3)
C4—C5—C6—C7	1.6 (5)	C22—C23—C28—N6	−8.1 (4)
F1—C6—C7—C8	−177.3 (3)	C22—C21—C29—N6	−6.4 (5)
C5—C6—C7—C8	−1.3 (6)	C20—C21—C29—N6	175.8 (3)
F1—C6—C7—N3	−1.5 (5)	N6—C30—C31—C32	106.8 (3)
C5—C6—C7—N3	174.6 (4)	N6—C30—C32—C31	−108.5 (3)
N3—C7—C8—F2	13.7 (6)	N7—C33—C34—N8	−54.7 (4)
C6—C7—C8—F2	−170.5 (3)	N7—C33—C34—C37	−174.6 (3)
N3—C7—C8—C9	−170.5 (4)	N8—C35—C36—N7	51.8 (3)
C6—C7—C8—C9	5.3 (5)	C38—C35—C36—N7	170.3 (3)
F2—C8—C9—N2	−13.1 (5)	C21—C29—N6—C28	−5.1 (5)
C7—C8—C9—N2	171.2 (3)	C21—C29—N6—C30	169.6 (3)
F2—C8—C9—C4	166.2 (3)	C27—C28—N6—C29	−166.3 (3)
C7—C8—C9—C4	−9.6 (5)	C23—C28—N6—C29	12.2 (4)
C5—C4—C9—C8	9.4 (5)	C27—C28—N6—C30	19.2 (5)
C3—C4—C9—C8	−169.0 (3)	C23—C28—N6—C30	−162.3 (3)
C5—C4—C9—N2	−171.3 (3)	C31—C30—N6—C29	−116.4 (3)
C3—C4—C9—N2	10.2 (4)	C32—C30—N6—C29	−47.3 (4)
C3—C2—C10—N2	6.6 (5)	C31—C30—N6—C28	58.1 (4)
C1—C2—C10—N2	−176.6 (3)	C32—C30—N6—C28	127.3 (3)
N2—C11—C12—C13	−106.1 (3)	C25—C26—N7—C33	−37.3 (5)
N2—C11—C13—C12	109.6 (3)	C27—C26—N7—C33	141.8 (4)
N3—C14—C15—N4	−51.3 (4)	C25—C26—N7—C36	127.9 (4)
N3—C14—C15—C18	−174.7 (3)	C27—C26—N7—C36	−52.9 (5)
N4—C16—C17—N3	56.3 (5)	C34—C33—N7—C26	−135.1 (3)
C19—C16—C17—N3	179.7 (3)	C34—C33—N7—C36	58.6 (4)
C2—C10—N2—C9	2.2 (5)	C35—C36—N7—C26	134.6 (3)
C2—C10—N2—C11	−171.9 (3)	C35—C36—N7—C33	−58.8 (4)
C8—C9—N2—C10	168.7 (3)	C33—C34—N8—C35	54.3 (3)
C4—C9—N2—C10	−10.6 (4)	C37—C34—N8—C35	176.9 (3)
C8—C9—N2—C11	−17.4 (5)	C36—C35—N8—C34	−51.8 (3)
C4—C9—N2—C11	163.4 (3)	C38—C35—N8—C34	−172.3 (3)
C12—C11—N2—C10	114.2 (3)	O4—C20—O5—Mn1	−173.4 (3)
C13—C11—N2—C10	45.1 (4)	C21—C20—O5—Mn1	5.1 (6)
C12—C11—N2—C9	−59.7 (4)	O2—Mn1—O5—C20	−148.0 (4)
C13—C11—N2—C9	−128.8 (3)	O3—Mn1—O5—C20	−67.6 (5)
C8—C7—N3—C14	−129.4 (4)	O6—Mn1—O5—C20	−6.4 (4)
C6—C7—N3—C14	55.0 (6)	O7—Mn1—O5—C20	127.3 (4)
C8—C7—N3—C17	30.0 (6)	O8—Mn1—O5—C20	75.3 (4)
C6—C7—N3—C17	−145.7 (4)	C21—C22—O6—Mn1	−11.2 (4)
C15—C14—N3—C7	−138.7 (4)	C23—C22—O6—Mn1	167.89 (19)
C15—C14—N3—C17	60.5 (5)	O5—Mn1—O6—C22	9.3 (3)
C16—C17—N3—C7	135.3 (4)	O2—Mn1—O6—C22	96.8 (3)
C16—C17—N3—C14	−63.0 (5)	O3—Mn1—O6—C22	168.7 (3)
C17—C16—N4—C15	−50.3 (4)	O7—Mn1—O6—C22	−70.2 (3)
C19—C16—N4—C15	−175.9 (3)	O8—Mn1—O6—C22	−104.8 (3)
C14—C15—N4—C16	47.4 (4)	C44—C39—C40—C41	0.4 (5)
C18—C15—N4—C16	174.3 (3)	C45—C39—C40—C41	179.8 (3)
O1—C1—O2—Mn1	163.6 (3)	C39—C40—C41—C42	0.5 (5)
C2—C1—O2—Mn1	−16.4 (5)	C40—C41—C42—C43	−0.2 (5)
O5—Mn1—O2—C1	171.9 (4)	C40—C41—C42—C46	178.7 (3)
O3—Mn1—O2—C1	15.5 (4)	C41—C42—C43—C44	−0.9 (5)
O6—Mn1—O2—C1	88.8 (4)	C46—C42—C43—C44	−179.8 (3)
O7—Mn1—O2—C1	−100.8 (4)	C42—C43—C44—C39	1.7 (6)
O8—Mn1—O2—C1	−62.8 (4)	C40—C39—C44—C43	−1.4 (5)
C2—C3—O3—Mn1	7.0 (4)	C45—C39—C44—C43	179.1 (3)
C4—C3—O3—Mn1	−172.56 (19)	C44—C39—C45—O8	176.4 (3)
O5—Mn1—O3—C3	−93.1 (3)	C40—C39—C45—O8	−3.1 (5)
O2—Mn1—O3—C3	−10.0 (3)	C44—C39—C45—O7	−4.8 (5)
O6—Mn1—O3—C3	−153.5 (3)	C40—C39—C45—O7	175.7 (3)
O7—Mn1—O3—C3	70.5 (3)	C43—C42—C46—O10	−156.2 (4)
O8—Mn1—O3—C3	120.7 (3)	C41—C42—C46—O10	24.9 (5)
O4—C20—C21—C29	−7.0 (4)	C43—C42—C46—O9	25.0 (5)
O5—C20—C21—C29	174.4 (3)	C41—C42—C46—O9	−153.9 (4)
O4—C20—C21—C22	175.4 (3)	O8—C45—O7—Mn1	−0.2 (3)
O5—C20—C21—C22	−3.1 (5)	C39—C45—O7—Mn1	−179.0 (2)
C29—C21—C22—O6	−171.0 (3)	O5—Mn1—O7—C45	−119.6 (2)
C20—C21—C22—O6	6.6 (5)	O2—Mn1—O7—C45	145.4 (2)
C29—C21—C22—C23	9.9 (4)	O3—Mn1—O7—C45	66.9 (2)
C20—C21—C22—C23	−172.6 (3)	O6—Mn1—O7—C45	−42.6 (2)
O6—C22—C23—C28	178.0 (3)	O8—Mn1—O7—C45	0.11 (18)
C21—C22—C23—C28	−2.8 (4)	O7—C45—O8—Mn1	0.2 (3)
O6—C22—C23—C24	−5.5 (4)	C39—C45—O8—Mn1	179.0 (3)
C21—C22—C23—C24	173.7 (3)	O5—Mn1—O8—C45	70.51 (19)
C28—C23—C24—N5	−172.9 (3)	O2—Mn1—O8—C45	−47.3 (2)
C22—C23—C24—N5	10.5 (4)	O3—Mn1—O8—C45	−123.57 (18)
C28—C23—C24—C25	3.5 (4)	O6—Mn1—O8—C45	149.90 (18)
C22—C23—C24—C25	−173.1 (3)	O7—Mn1—O8—C45	−0.11 (18)

### (I) 0.25-Aqua(benzene-1,4-dicarboxylato-κ2O,O')bis(sparfloxacin-κ2O,O')manganese(II) dihydrate Hydrogen-bond geometry (Å, º)

Cg9 is the centroid of the C39–C44 ring.

**Table:**

*D*—H···*A*	*D*—H	H···*A*	*D*···*A*	*D*—H···*A*
N1—H1*A*···O8^i^	0.86	2.24	3.041 (3)	156
N1—H1*B*···O3	0.86	2.02	2.651 (3)	129
N4—H4*A*···O1^ii^	0.90	1.80	2.647 (4)	156
N4—H4*B*···O11^iii^	0.90	2.01	2.845 (3)	153
N5—H5*A*···O8^i^	0.86	2.13	2.954 (3)	160
N5—H5*B*···O6	0.86	2.01	2.651 (3)	131
N8—H8*A*···O4^iv^	0.90	1.85	2.750 (3)	175
N8—H8*B*···O11^v^	0.90	1.94	2.821 (3)	166
O11—H1*W*···O9^vi^	0.85	1.76	2.606 (3)	174
O11—H2*W*···O7^vii^	0.85	1.97	2.804 (3)	171
O12—H3*W*···O10^i^	0.85	2.11	2.924 (4)	159
O12—H4*W*···O10^vi^	0.85	2.00	2.844 (4)	174
O13—H5*W*···O2^vii^	0.95	2.41	3.000 (12)	120
C13—H13*B*···O4^vii^	0.97	2.56	3.526 (5)	178
C35—H35···O12^v^	0.98	2.38	3.321 (4)	161
C38—H38*A*···O13^v^	0.96	2.51	3.097 (12)	120
C12—H12*A*···*Cg*9^viii^	0.97	2.59	3.529 (4)	162

Symmetry codes: (i) −*x*, −*y*+1, −*z*; (ii) *x*−1/2, −*y*+1/2, *z*+1/2; (iii) −*x*+1/2, *y*−1/2, −*z*+1/2; (iv) *x*−1/2, −*y*+3/2, *z*+1/2; (v) −*x*+1/2, *y*+1/2, −*z*+1/2; (vi) *x*+1, *y*, *z*+1; (vii) −*x*+1, −*y*+1, −*z*; (viii) *x*+1/2, −*y*+1/2, *z*+1/2.

### (II) Bis(sparfloxacin-κ2O,O')copper(II) benzene-1,4-dicarboxylate dihydrate Crystal data

**Table:**

[Cu(C_19_H_22_F_2_N_4_O_3_)_2_](C_8_H_4_O_4_)·2H_2_O	*F*(000) = 1090
*M**_r_* = 1048.50	*D*_x_ = 1.529 Mg m^−^^3^
Monoclinic, *P*2_1_/*c*	Mo *K*α radiation, λ = 0.71073 Å
Hall symbol: -P 2ybc	Cell parameters from 3693 reflections
*a* = 13.6039 (2) Å	θ = 2.7–26.5°
*b* = 7.8019 (1) Å	µ = 0.57 mm^−^^1^
*c* = 22.0870 (3) Å	*T* = 296 K
β = 103.764 (1)°	Block, green
*V* = 2276.91 (5) Å^3^	0.20 × 0.17 × 0.13 mm
*Z* = 2	

### (II) Bis(sparfloxacin-κ2O,O')copper(II) benzene-1,4-dicarboxylate dihydrate Data collection

**Table:**

Bruker SMART CCD diffractometer	5168 independent reflections
Radiation source: fine-focus sealed tube	3641 reflections with *I* > 2σ(*I*)
Graphite monochromator	*R*_int_ = 0.049
ω scans	θ_max_ = 27.4°, θ_min_ = 2.7°
Absorption correction: multi-scan (SADABS; Bruker, 2004)	*h* = −17→17
*T*_min_ = 0.895, *T*_max_ = 0.930	*k* = −10→10
20662 measured reflections	*l* = −28→23

### (II) Bis(sparfloxacin-κ2O,O')copper(II) benzene-1,4-dicarboxylate dihydrate Refinement

**Table:**

Refinement on *F*^2^	Primary atom site location: structure-invariant direct methods
Least-squares matrix: full	Secondary atom site location: difference Fourier map
*R*[*F*^2^ > 2σ(*F*^2^)] = 0.052	Hydrogen site location: inferred from neighbouring sites
*wR*(*F*^2^) = 0.142	H-atom parameters constrained
*S* = 1.06	*w* = 1/[σ^2^(*F*_o_^2^) + (0.0611*P*)^2^ + 1.3659*P*] where *P* = (*F*_o_^2^ + 2*F*_c_^2^)/3
5168 reflections	(Δ/σ)_max_ < 0.001
323 parameters	Δρ_max_ = 0.56 e Å^−^^3^
0 restraints	Δρ_min_ = −0.57 e Å^−^^3^

### (II) Bis(sparfloxacin-κ2O,O')copper(II) benzene-1,4-dicarboxylate dihydrate Special details

**Table:**

Geometry. All esds (except the esd in the dihedral angle between two l.s. planes) are estimated using the full covariance matrix. The cell esds are taken into account individually in the estimation of esds in distances, angles and torsion angles; correlations between esds in cell parameters are only used when they are defined by crystal symmetry. An approximate (isotropic) treatment of cell esds is used for estimating esds involving l.s. planes.
Refinement. Refinement of F^2^ against ALL reflections. The weighted R-factor wR and goodness of fit S are based on F^2^, conventional R-factors R are based on F, with F set to zero for negative F^2^. The threshold expression of F^2^ > 2sigma(F^2^) is used only for calculating R-factors(gt) etc. and is not relevant to the choice of reflections for refinement. R-factors based on F^2^ are statistically about twice as large as those based on F, and R- factors based on ALL data will be even larger.

### (II) Bis(sparfloxacin-κ2O,O')copper(II) benzene-1,4-dicarboxylate dihydrate Fractional atomic coordinates and isotropic or equivalent isotropic displacement parameters (Å^2^)

**Table:**

	*x*	*y*	*z*	*U*_iso_*/*U*_eq_	Occ. (<1)
Cu1	0.5000	0.0000	0.5000	0.03589 (15)	
C1	0.4364 (3)	0.1538 (4)	0.60336 (15)	0.0509 (8)	
C2	0.3912 (2)	0.3007 (4)	0.56214 (13)	0.0406 (7)	
C3	0.4015 (2)	0.3268 (3)	0.50090 (13)	0.0344 (6)	
C4	0.3610 (2)	0.4832 (3)	0.46921 (13)	0.0344 (6)	
C5	0.3720 (2)	0.5207 (3)	0.40786 (13)	0.0378 (6)	
C6	0.3248 (2)	0.6682 (4)	0.37975 (14)	0.0436 (7)	
C7	0.2699 (2)	0.7814 (4)	0.40656 (14)	0.0439 (7)	
C8	0.2666 (3)	0.7474 (4)	0.46785 (14)	0.0521 (8)	
C9	0.3090 (2)	0.6018 (4)	0.49971 (13)	0.0428 (7)	
C10	0.3449 (3)	0.4244 (4)	0.58938 (15)	0.0527 (8)	
H10	0.3410	0.4065	0.6304	0.063*	
C11A	0.3034 (7)	0.7255 (11)	0.5989 (4)	0.034 (2)*	0.330 (8)
H11A	0.3558	0.8116	0.5986	0.041*	0.330 (8)
C11B	0.2443 (4)	0.6775 (6)	0.5977 (2)	0.0414 (13)*	0.670 (8)
H11B	0.1714	0.6862	0.5796	0.050*	0.670 (8)
C12	0.2750 (4)	0.6913 (5)	0.6618 (2)	0.0779 (13)	
H12A	0.3388	0.6498	0.6871	0.093*	
H12B	0.2167	0.6809	0.6797	0.093*	
C13A	0.1996 (8)	0.7884 (13)	0.5963 (5)	0.050 (3)*	0.330 (8)
H13A	0.1294	0.7534	0.5824	0.060*	0.330 (8)
H13B	0.2141	0.9063	0.5875	0.060*	0.330 (8)
C13B	0.2951 (4)	0.8341 (7)	0.6235 (2)	0.0548 (16)*	0.670 (8)
H13C	0.2498	0.9319	0.6178	0.066*	0.670 (8)
H13D	0.3652	0.8611	0.6250	0.066*	0.670 (8)
C14	0.2694 (2)	1.0478 (4)	0.34509 (16)	0.0477 (8)	
H14A	0.3360	1.0072	0.3431	0.057*	
H14B	0.2780	1.1508	0.3704	0.057*	
C15	0.2084 (2)	1.0892 (4)	0.28020 (14)	0.0399 (6)	
H15	0.2049	0.9859	0.2545	0.048*	
C16	0.0536 (2)	1.0046 (4)	0.31469 (13)	0.0388 (6)	
H16	0.0481	0.8983	0.2905	0.047*	
C17	0.1190 (2)	0.9702 (4)	0.37892 (14)	0.0453 (7)	
H17A	0.1240	1.0729	0.4042	0.054*	
H17B	0.0890	0.8802	0.3990	0.054*	
C18	0.2539 (3)	1.2321 (5)	0.24928 (16)	0.0561 (9)	
H18A	0.2150	1.2467	0.2072	0.084*	
H18B	0.3224	1.2035	0.2490	0.084*	
H18C	0.2532	1.3367	0.2720	0.084*	
C19	−0.0522 (2)	1.0637 (5)	0.31529 (17)	0.0557 (8)	
H19A	−0.0484	1.1682	0.3387	0.084*	
H19B	−0.0852	0.9771	0.3342	0.084*	
H19C	−0.0902	1.0833	0.2733	0.084*	
N1	0.4226 (2)	0.4198 (3)	0.37568 (12)	0.0482 (6)	
H1A	0.4252	0.4473	0.3384	0.058*	
H1B	0.4521	0.3281	0.3925	0.058*	
N2	0.3048 (2)	0.5684 (3)	0.56173 (11)	0.0517 (7)	
N3	0.2191 (2)	0.9183 (3)	0.37340 (13)	0.0519 (7)	
N4	0.10343 (17)	1.1367 (3)	0.28308 (10)	0.0355 (5)	
H4A	0.0659	1.1525	0.2440	0.043*	
H4B	0.1053	1.2368	0.3036	0.043*	
O1	0.4311 (3)	0.1573 (4)	0.65800 (12)	0.0909 (11)	
O2	0.47895 (16)	0.0304 (2)	0.58081 (9)	0.0447 (5)	
O3	0.44457 (16)	0.2185 (2)	0.47204 (9)	0.0438 (5)	
F1	0.32798 (15)	0.6955 (2)	0.31954 (9)	0.0609 (5)	
F2A	0.2432 (5)	0.8883 (6)	0.49417 (18)	0.0451 (16)*	0.456 (11)
F2B	0.1995 (4)	0.8500 (5)	0.49550 (15)	0.0436 (13)*	0.544 (11)
C20	−0.0172 (2)	0.0098 (3)	0.06024 (12)	0.0350 (6)	
C21	0.0523 (2)	0.1177 (4)	0.04203 (13)	0.0416 (7)	
H21	0.0877	0.1977	0.0701	0.050*	
C22	0.0694 (2)	0.1077 (4)	−0.01728 (13)	0.0420 (7)	
H22	0.1163	0.1805	−0.0284	0.050*	
C23	−0.0320 (2)	0.0165 (4)	0.12619 (13)	0.0415 (7)	
O4	−0.0936 (2)	−0.0847 (3)	0.14030 (11)	0.0607 (6)	
O5	0.02068 (19)	0.1251 (3)	0.16135 (10)	0.0598 (6)	
O6	0.4982 (2)	0.0203 (4)	0.23468 (11)	0.0704 (7)	
H1W	0.5212	0.0005	0.2729	0.084*	
H2W	0.5047	0.1270	0.2369	0.084*	

### (II) Bis(sparfloxacin-κ2O,O')copper(II) benzene-1,4-dicarboxylate dihydrate Atomic displacement parameters (Å^2^)

**Table:**

	*U*^11^	*U*^22^	*U*^33^	*U*^12^	*U*^13^	*U*^23^
Cu1	0.0406 (3)	0.0292 (2)	0.0378 (3)	0.0048 (2)	0.0093 (2)	0.0081 (2)
C1	0.067 (2)	0.0454 (17)	0.0404 (18)	0.0155 (16)	0.0134 (16)	0.0122 (14)
C2	0.0490 (17)	0.0345 (14)	0.0355 (16)	0.0092 (13)	0.0048 (13)	0.0046 (12)
C3	0.0367 (14)	0.0278 (13)	0.0360 (15)	0.0007 (11)	0.0029 (12)	0.0022 (11)
C4	0.0380 (14)	0.0273 (13)	0.0336 (14)	0.0008 (11)	0.0001 (11)	0.0034 (11)
C5	0.0404 (15)	0.0316 (14)	0.0383 (15)	0.0003 (12)	0.0035 (12)	0.0055 (11)
C6	0.0482 (17)	0.0397 (15)	0.0403 (17)	−0.0002 (13)	0.0052 (13)	0.0144 (13)
C7	0.0517 (18)	0.0300 (14)	0.0427 (17)	−0.0012 (13)	−0.0034 (14)	0.0051 (12)
C8	0.072 (2)	0.0357 (15)	0.0409 (18)	0.0212 (15)	−0.0020 (16)	−0.0050 (13)
C9	0.0573 (18)	0.0327 (14)	0.0319 (15)	0.0084 (13)	−0.0021 (13)	0.0008 (12)
C10	0.074 (2)	0.0498 (18)	0.0316 (16)	0.0214 (17)	0.0065 (15)	0.0055 (14)
C12	0.121 (4)	0.060 (2)	0.071 (3)	0.025 (2)	0.060 (3)	0.016 (2)
C14	0.0425 (16)	0.0394 (16)	0.057 (2)	0.0035 (13)	0.0037 (15)	0.0146 (14)
C15	0.0419 (16)	0.0387 (15)	0.0394 (16)	0.0073 (13)	0.0107 (13)	−0.0013 (12)
C16	0.0413 (15)	0.0351 (14)	0.0384 (15)	0.0018 (13)	0.0061 (12)	−0.0007 (12)
C17	0.0554 (18)	0.0365 (16)	0.0419 (17)	0.0027 (13)	0.0073 (14)	0.0069 (12)
C18	0.0535 (19)	0.066 (2)	0.052 (2)	0.0078 (17)	0.0185 (16)	0.0159 (17)
C19	0.0435 (18)	0.0561 (19)	0.068 (2)	0.0041 (15)	0.0139 (16)	0.0055 (17)
N1	0.0643 (17)	0.0419 (14)	0.0408 (15)	0.0110 (13)	0.0172 (13)	0.0104 (11)
N2	0.0762 (19)	0.0417 (13)	0.0320 (14)	0.0241 (14)	0.0029 (13)	−0.0006 (11)
N3	0.0474 (15)	0.0411 (14)	0.0632 (18)	0.0071 (12)	0.0052 (13)	0.0232 (13)
N4	0.0418 (13)	0.0366 (12)	0.0263 (11)	0.0080 (10)	0.0042 (10)	−0.0001 (9)
O1	0.152 (3)	0.0843 (19)	0.0448 (15)	0.068 (2)	0.0403 (17)	0.0296 (14)
O2	0.0549 (13)	0.0389 (11)	0.0418 (12)	0.0145 (9)	0.0142 (10)	0.0134 (9)
O3	0.0609 (13)	0.0330 (10)	0.0387 (11)	0.0122 (9)	0.0142 (10)	0.0085 (8)
F1	0.0745 (13)	0.0592 (12)	0.0513 (12)	0.0185 (10)	0.0193 (10)	0.0265 (9)
C20	0.0431 (15)	0.0351 (14)	0.0260 (13)	0.0060 (12)	0.0066 (11)	0.0048 (11)
C21	0.0536 (17)	0.0391 (15)	0.0287 (15)	−0.0068 (13)	0.0032 (13)	−0.0030 (12)
C22	0.0532 (18)	0.0396 (15)	0.0336 (16)	−0.0070 (13)	0.0110 (13)	0.0041 (12)
C23	0.0573 (18)	0.0387 (15)	0.0281 (15)	0.0126 (14)	0.0097 (13)	0.0064 (12)
O4	0.0901 (18)	0.0541 (14)	0.0470 (13)	−0.0027 (13)	0.0346 (13)	0.0105 (11)
O5	0.0852 (18)	0.0659 (15)	0.0255 (11)	−0.0022 (13)	0.0072 (11)	−0.0046 (11)
O6	0.0863 (19)	0.0776 (18)	0.0439 (14)	0.0018 (15)	0.0089 (13)	0.0074 (12)

### (II) Bis(sparfloxacin-κ2O,O')copper(II) benzene-1,4-dicarboxylate dihydrate Geometric parameters (Å, º)

**Table:**

Cu1—O2	1.889 (2)	C13A—H13B	0.9700
Cu1—O2^i^	1.889 (2)	C13B—H13C	0.9700
Cu1—O3^i^	1.9064 (18)	C13B—H13D	0.9700
Cu1—O3	1.9064 (18)	C14—N3	1.444 (4)
C1—O1	1.226 (4)	C14—C15	1.510 (4)
C1—O2	1.283 (4)	C14—H14A	0.9700
C1—C2	1.501 (4)	C14—H14B	0.9700
C2—C10	1.368 (4)	C15—N4	1.492 (3)
C2—C3	1.407 (4)	C15—C18	1.515 (4)
C3—O3	1.281 (3)	C15—H15	0.9800
C3—C4	1.449 (4)	C16—N4	1.494 (4)
C4—C9	1.428 (4)	C16—C17	1.508 (4)
C4—C5	1.428 (4)	C16—C19	1.515 (4)
C5—N1	1.353 (4)	C16—H16	0.9800
C5—C6	1.390 (4)	C17—N3	1.454 (4)
C6—F1	1.358 (3)	C17—H17A	0.9700
C6—C7	1.377 (4)	C17—H17B	0.9700
C7—N3	1.384 (4)	C18—H18A	0.9600
C7—C8	1.391 (4)	C18—H18B	0.9600
C8—F2A	1.317 (5)	C18—H18C	0.9600
C8—C9	1.387 (4)	C19—H19A	0.9600
C8—F2B	1.453 (5)	C19—H19B	0.9600
C9—N2	1.409 (4)	C19—H19C	0.9600
C10—N2	1.332 (4)	N1—H1A	0.8600
C10—H10	0.9300	N1—H1B	0.8600
C11A—N2	1.478 (9)	N4—H4A	0.9000
C11A—C13A	1.483 (14)	N4—H4B	0.9000
C11A—C12	1.551 (9)	C20—C22^ii^	1.387 (4)
C11A—H11A	0.9800	C20—C21	1.395 (4)
C11B—C12	1.380 (6)	C20—C23	1.518 (4)
C11B—C13B	1.451 (7)	C21—C22	1.386 (4)
C11B—N2	1.532 (5)	C21—H21	0.9300
C11B—H11B	0.9800	C22—C20^ii^	1.387 (4)
C12—C13B	1.463 (6)	C22—H22	0.9300
C12—C13A	1.736 (11)	C23—O4	1.244 (4)
C12—H12A	0.9700	C23—O5	1.253 (4)
C12—H12B	0.9700	O6—H1W	0.8412
C13A—H13A	0.9700	O6—H2W	0.8376
			
O2—Cu1—O2^i^	180.0	C15—C14—H14A	109.5
O2—Cu1—O3^i^	86.76 (8)	N3—C14—H14B	109.5
O2^i^—Cu1—O3^i^	93.24 (8)	C15—C14—H14B	109.5
O2—Cu1—O3	93.24 (8)	H14A—C14—H14B	108.1
O2^i^—Cu1—O3	86.76 (8)	N4—C15—C14	109.1 (2)
O3^i^—Cu1—O3	180.0	N4—C15—C18	109.5 (2)
O1—C1—O2	122.4 (3)	C14—C15—C18	113.2 (3)
O1—C1—C2	117.9 (3)	N4—C15—H15	108.3
O2—C1—C2	119.6 (3)	C14—C15—H15	108.3
C10—C2—C3	118.8 (3)	C18—C15—H15	108.3
C10—C2—C1	115.9 (3)	N4—C16—C17	109.3 (2)
C3—C2—C1	125.0 (3)	N4—C16—C19	109.4 (2)
O3—C3—C2	123.0 (2)	C17—C16—C19	113.3 (3)
O3—C3—C4	118.8 (2)	N4—C16—H16	108.3
C2—C3—C4	118.3 (2)	C17—C16—H16	108.3
C9—C4—C5	119.4 (2)	C19—C16—H16	108.3
C9—C4—C3	119.5 (2)	N3—C17—C16	109.0 (3)
C5—C4—C3	121.2 (2)	N3—C17—H17A	109.9
N1—C5—C6	119.0 (3)	C16—C17—H17A	109.9
N1—C5—C4	124.1 (2)	N3—C17—H17B	109.9
C6—C5—C4	116.9 (3)	C16—C17—H17B	109.9
F1—C6—C7	117.9 (2)	H17A—C17—H17B	108.3
F1—C6—C5	116.6 (3)	C15—C18—H18A	109.5
C7—C6—C5	125.3 (3)	C15—C18—H18B	109.5
C6—C7—N3	121.4 (3)	H18A—C18—H18B	109.5
C6—C7—C8	116.3 (3)	C15—C18—H18C	109.5
N3—C7—C8	122.3 (3)	H18A—C18—H18C	109.5
F2A—C8—C9	125.0 (3)	H18B—C18—H18C	109.5
F2A—C8—C7	109.7 (3)	C16—C19—H19A	109.5
C9—C8—C7	123.0 (3)	C16—C19—H19B	109.5
F2A—C8—F2B	27.5 (2)	H19A—C19—H19B	109.5
C9—C8—F2B	117.7 (3)	C16—C19—H19C	109.5
C7—C8—F2B	118.2 (3)	H19A—C19—H19C	109.5
C8—C9—N2	122.5 (3)	H19B—C19—H19C	109.5
C8—C9—C4	119.0 (3)	C5—N1—H1A	120.0
N2—C9—C4	118.6 (2)	C5—N1—H1B	120.0
N2—C10—C2	125.1 (3)	H1A—N1—H1B	120.0
N2—C10—H10	117.5	C10—N2—C9	119.7 (3)
C2—C10—H10	117.5	C10—N2—C11A	120.2 (4)
N2—C11A—C13A	113.0 (7)	C9—N2—C11A	113.3 (4)
N2—C11A—C12	113.2 (6)	C10—N2—C11B	116.3 (3)
C13A—C11A—C12	69.7 (6)	C9—N2—C11B	123.6 (3)
N2—C11A—H11A	117.4	C11A—N2—C11B	34.0 (3)
C13A—C11A—H11A	117.4	C7—N3—C14	122.8 (3)
C12—C11A—H11A	117.4	C7—N3—C17	122.1 (3)
C13A—C11A—H13D	104.0	C14—N3—C17	113.0 (2)
C12—C11B—C13B	62.2 (3)	C15—N4—C16	113.2 (2)
C12—C11B—N2	120.4 (4)	C15—N4—H4A	108.9
C13B—C11B—N2	114.0 (4)	C16—N4—H4A	108.9
C12—C11B—H11B	116.3	C15—N4—H4B	108.9
C13B—C11B—H11B	116.3	C16—N4—H4B	108.9
N2—C11B—H11B	116.3	H4A—N4—H4B	107.8
C11B—C12—C13B	61.3 (3)	C1—O2—Cu1	130.16 (19)
C13B—C12—H12A	108.8	C3—O3—Cu1	128.39 (18)
C11A—C12—H12A	101.2	F2B—F2A—C8	87.6 (6)
C11B—C12—H12B	109.5	F2A—F2B—C8	64.9 (5)
H12A—C12—H12B	116.3	C22^ii^—C20—C21	118.3 (3)
C11A—C13A—C12	57.0 (5)	C22^ii^—C20—C23	121.1 (3)
C11A—C13A—H13A	140.9	C21—C20—C23	120.6 (3)
C12—C13A—H13A	118.8	C22—C21—C20	121.1 (3)
C12—C13A—H13B	118.2	C22—C21—H21	119.5
H13A—C13A—H13B	115.8	C20—C21—H21	119.5
C11B—C13B—C12	56.5 (3)	C21—C22—C20^ii^	120.7 (3)
C12—C13B—H13B	115.4	C21—C22—H22	119.7
C11B—C13B—H13C	112.6	C20^ii^—C22—H22	119.7
C12—C13B—H13C	118.2	O4—C23—O5	126.4 (3)
C12—C13B—H13D	117.6	O4—C23—C20	118.0 (3)
H13C—C13B—H13D	115.0	O5—C23—C20	115.6 (3)
N3—C14—C15	110.5 (3)	H1W—O6—H2W	96.7
N3—C14—H14A	109.5		
			
O1—C1—C2—C10	−2.0 (5)	C13A—C12—C13B—C11B	−43.5 (5)
O2—C1—C2—C10	178.2 (3)	N3—C14—C15—N4	54.3 (3)
O1—C1—C2—C3	171.9 (3)	N3—C14—C15—C18	176.5 (3)
O2—C1—C2—C3	−7.9 (5)	N4—C16—C17—N3	−56.5 (3)
C10—C2—C3—O3	179.4 (3)	C19—C16—C17—N3	−178.7 (3)
C1—C2—C3—O3	5.7 (5)	C2—C10—N2—C9	−0.2 (6)
C10—C2—C3—C4	−0.8 (4)	C2—C10—N2—C11A	−149.3 (5)
C1—C2—C3—C4	−174.5 (3)	C2—C10—N2—C11B	172.1 (4)
O3—C3—C4—C9	177.4 (3)	C8—C9—N2—C10	178.4 (3)
C2—C3—C4—C9	−2.5 (4)	C4—C9—N2—C10	−3.2 (5)
O3—C3—C4—C5	−2.4 (4)	C8—C9—N2—C11A	−30.4 (6)
C2—C3—C4—C5	177.8 (3)	C4—C9—N2—C11A	148.0 (4)
C9—C4—C5—N1	177.9 (3)	C8—C9—N2—C11B	6.7 (5)
C3—C4—C5—N1	−2.4 (4)	C4—C9—N2—C11B	−174.9 (3)
C9—C4—C5—C6	−3.9 (4)	C13A—C11A—N2—C10	−118.7 (6)
C3—C4—C5—C6	175.8 (3)	C12—C11A—N2—C10	−41.9 (8)
N1—C5—C6—F1	3.8 (4)	C13A—C11A—N2—C9	90.3 (7)
C4—C5—C6—F1	−174.5 (2)	C12—C11A—N2—C9	167.2 (4)
N1—C5—C6—C7	179.2 (3)	C13A—C11A—N2—C11B	−25.7 (6)
C4—C5—C6—C7	0.9 (4)	C12—C11A—N2—C11B	51.2 (6)
F1—C6—C7—N3	0.3 (4)	C12—C11B—N2—C10	36.8 (6)
C5—C6—C7—N3	−175.0 (3)	C13B—C11B—N2—C10	107.5 (4)
F1—C6—C7—C8	178.7 (3)	C12—C11B—N2—C9	−151.2 (4)
C5—C6—C7—C8	3.4 (5)	C13B—C11B—N2—C9	−80.5 (5)
C6—C7—C8—F2A	158.9 (4)	C12—C11B—N2—C11A	−69.0 (7)
N3—C7—C8—F2A	−22.7 (5)	C13B—C11B—N2—C11A	1.7 (6)
C6—C7—C8—C9	−4.7 (5)	C6—C7—N3—C14	−58.0 (4)
N3—C7—C8—C9	173.6 (3)	C8—C7—N3—C14	123.7 (4)
C6—C7—C8—F2B	−172.4 (4)	C6—C7—N3—C17	139.8 (3)
N3—C7—C8—F2B	6.0 (5)	C8—C7—N3—C17	−38.5 (4)
F2A—C8—C9—N2	19.1 (7)	C15—C14—N3—C7	136.4 (3)
C7—C8—C9—N2	−179.8 (3)	C15—C14—N3—C17	−60.0 (4)
F2B—C8—C9—N2	−12.1 (5)	C16—C17—N3—C7	−135.5 (3)
F2A—C8—C9—C4	−159.2 (5)	C16—C17—N3—C14	60.8 (3)
C7—C8—C9—C4	1.8 (5)	C14—C15—N4—C16	−53.9 (3)
F2B—C8—C9—C4	169.5 (3)	C18—C15—N4—C16	−178.3 (2)
C5—C4—C9—C8	2.7 (4)	C17—C16—N4—C15	55.5 (3)
C3—C4—C9—C8	−177.1 (3)	C19—C16—N4—C15	−179.9 (2)
C5—C4—C9—N2	−175.8 (3)	O1—C1—O2—Cu1	−177.8 (3)
C3—C4—C9—N2	4.5 (4)	C2—C1—O2—Cu1	2.0 (5)
C3—C2—C10—N2	2.2 (5)	O3^i^—Cu1—O2—C1	−176.4 (3)
C1—C2—C10—N2	176.5 (3)	O3—Cu1—O2—C1	3.6 (3)
N2—C11B—C12—C13B	102.9 (5)	C2—C3—O3—Cu1	2.4 (4)
C13B—C11B—C12—C11A	−41.2 (6)	C4—C3—O3—Cu1	−177.49 (18)
N2—C11B—C12—C11A	61.7 (7)	O2—Cu1—O3—C3	−6.0 (2)
C13B—C11B—C12—C13A	58.5 (7)	O2^i^—Cu1—O3—C3	174.0 (2)
N2—C11B—C12—C13A	161.5 (9)	C9—C8—F2A—F2B	−83.2 (6)
N2—C11A—C12—C11B	−58.9 (6)	C7—C8—F2A—F2B	113.6 (5)
C13A—C11A—C12—C11B	48.2 (6)	C9—C8—F2B—F2A	113.3 (6)
N2—C11A—C12—C13B	−174.2 (10)	C7—C8—F2B—F2A	−78.4 (6)
C13A—C11A—C12—C13B	−67.1 (7)	C22^ii^—C20—C21—C22	−0.3 (5)
N2—C11A—C12—C13A	−107.1 (8)	C23—C20—C21—C22	177.2 (3)
N2—C11A—C13A—C12	107.4 (7)	C20—C21—C22—C20^ii^	0.3 (5)
C11B—C12—C13A—C11A	−44.0 (6)	C22^ii^—C20—C23—O4	−1.2 (4)
C13B—C12—C13A—C11A	51.5 (5)	C21—C20—C23—O4	−178.8 (3)
N2—C11B—C13B—C12	−113.1 (4)	C22^ii^—C20—C23—O5	178.5 (3)
C11A—C12—C13B—C11B	35.6 (6)	C21—C20—C23—O5	1.0 (4)

Symmetry codes: (i) −*x*+1, −*y*, −*z*+1; (ii) −*x*, −*y*, −*z*.

### (II) Bis(sparfloxacin-κ2O,O')copper(II) benzene-1,4-dicarboxylate dihydrate Hydrogen-bond geometry (Å, º)

**Table:**

*D*—H···*A*	*D*—H	H···*A*	*D*···*A*	*D*—H···*A*
N1—H1*A*···F1	0.86	2.33	2.657 (3)	103
N1—H1*A*···O6^iii^	0.86	2.19	2.995 (4)	155
N1—H1*B*···O3	0.86	1.98	2.604 (3)	129
N4—H4*A*···O5^iv^	0.90	1.80	2.658 (3)	160
N4—H4*B*···O4^v^	0.90	1.90	2.777 (3)	166
O6—H1*W*···O1^i^	0.84	1.95	2.716 (3)	151
O6—H2*W*···O1^vi^	0.84	2.46	3.048 (4)	128
C12—H12*A*···O6^vii^	0.97	2.55	3.494 (5)	166
C13*B*—H13*D*···O1^iv^	0.97	2.52	3.113 (6)	119
C13*B*—H13*D*···O2^iv^	0.97	2.41	3.258 (6)	146

Symmetry codes: (i) −*x*+1, −*y*, −*z*+1; (iii) −*x*+1, *y*+1/2, −*z*+1/2; (iv) *x*, *y*+1, *z*; (v) −*x*, *y*+3/2, −*z*+1/2; (vi) *x*, −*y*+1/2, *z*−1/2; (vii) *x*, −*y*+1/2, *z*+1/2.
